# Xenia Effect on Nutritional and Flavor Components of ‘Jingbaili’ Pear

**DOI:** 10.3390/foods14010094

**Published:** 2025-01-02

**Authors:** Yaxun Qiao, Wenjie Yu, Keju Li, Jingze Cao, Jie Zhu, Qiuning Wang, Jiaqi Zhao, Yunping Wang, Liping Luo, Jinwang Li, Fangjian Ning

**Affiliations:** Key Laboratory of Geriatric Nutrition and Health, School of Food and Health, Beijing Technology and Business University, Ministry of Education, Beijing 100048, China; 18713394920@163.com (Y.Q.); 2350201016@st.btbu.edu.cn (W.Y.); lkj5516@126.com (K.L.); caojingze_gsau@163.com (J.C.); 18830068270@163.com (J.Z.); 15901432273@163.com (Q.W.); zhaojaiqi0527@163.com (J.Z.); 19846950531@163.com (Y.W.); lluo2@126.com (L.L.); sdlijinwang@sina.com (J.L.)

**Keywords:** ‘Jingbaili’ pear, xenia effect, volatile compounds, metabolomics, fruit maturation, nutrient composition

## Abstract

The ‘Jingbaili’ pear is a national geographical indication product of China, featuring an oblate shape and being rich in nutrients. But the quality of the ‘Jingbaili’ pear is unstable. Xenia can cause changes in the quality of pears, but the effect of xenia on the ‘Jingbaili’ pear is unknown, and its mechanism is still unclear. In order to clarify the effect of pollination on the fruit quality of the ’Jingbaili’ pear, this research pollinated ‘Jingbaili’ pear flowers with the pollen of ‘Yali’ (JY), ‘Suli’ (JS) and ‘Huangli’ (JH). The results indicated that the mass, transverse diameter and longitudinal diameter of the JY group were significantly higher than the JS group and JH group. On the other hand, the pears of the JY group and JS group obtained higher soluble sugar content. The aroma content of characteristic compounds was higher in the JY group than in the JS group and JH group. Multivariate analysis revealed significant differences in the nonvolatile metabolites among the JY group, JS group and JH group, potentially explaining the variations in the nutritional and flavor compounds of the pears. Furthermore, this research investigated metabolic changes in the pears during development and ripening under the three types of pollination. The results showed that amino acid metabolism differed among these pollination types during development. These differences may be the cause of the observed variations in the pears. This research clarified the effect of xenia on the nutritional components and flavor substances in the ‘Jingbaili’ pear and could provide data support for improving the quality of the ‘Jingbaili’ pear.

## 1. Introduction

The ‘Jingbaili’ pear (*Pyrus ussuriensis* Maxim. cv. ‘Jingbaili’, JBP) is a highly regarded variety within the *Pyrus ussuriensis* species, which is well-liked by consumers due to its abundant nutritional content and robust flavor [1]. The JBP is native to Dongshan Village, Mentougou District, Beijing (GPS N 40.014°, E 116.135°), and is cultivated in Beijing, Hebei and Liaoning. In 2012, geographical indication product protection was implemented for the JBP. Pears (*Pyrus* spp.) are self-incompatible fruit trees, and fruit formation mainly relies on cross-pollination [2]. Therefore, selecting superior pollinating parents is crucial for obtaining high-quality pears. Nevertheless, the incorrect choice of pollinating varieties has resulted in a notable decline in the flavor and quality of the pears [3].

The influence that pollen has on maternal tissues, including the seed coat and pericarp, is termed xenia [3]. Xenia refers to the direct impact of pollen’s genetic composition on the diversity of seed and fruit development, as well as the physical characteristics that arise from fertilization until seed germination [4]. Hence, research on the xenia effect is of great significance to the production of fruit trees. Multiple studies have demonstrated that xenia exerts a substantial influence on fruit quality. Several investigations have shown that the quality of pomelo fruits pollinated by Citrus mangshanensis differed significantly from that of fruits resulting from natural pollination, indicating a xenia effect [5]. Similarly, the quality of ‘Yali’ pears was found to be significantly enhanced after pollination with ‘Hongnanguo’ and ‘Xuehuali’ pears [3]. Although the xenia effect can improve pear quality, studies on its impact on the quality of the JBP have not been reported. Furthermore, the effect of different pollen varieties on the quality of the JBP is unknown, and the exact mechanisms by which volatile compounds and metabolites are associated with the xenia effect remain unclear.

The quality and flavor of fruits are determined by metabolites such as sugars, organic acids, polyphenols and amino acids [6]. The sugar-acid fractions present in fruits are the fundamental components that constitute the flavor of the fruit. The specific types and amounts of soluble sugars and organic acids have a direct impact on the sweet and sour taste of fruits [7]. Polyphenols are crucial bioactive compounds and minerals found in fruits [8]. The composition and abundance of these chemicals serve as significant indicators for assessing fruit quality and defining taste.

Research has described that metabolite changes occurring in developing and ripening pears are similar to those reported previously, including changes in sugars, organic acids and some amino acids [9]. Furthermore, metabolomics was employed to investigate the impact of xenia effects on aroma quality in ‘Yali’ pears. After pollinating with ‘Hongnanguo’ pollen, the total aroma and ester content of ‘Yali’ pears were significantly higher than those of pears pollinated with ‘Xuehuali’ pollen [3]. The process of metabolites formation during fruit ripening is intricate, and there is a dearth of research on metabolites associated with the JBP.

In this research, ‘Yali’ (*Pyrus bretschneideri* Rehd. cv. ‘Yali’), ‘Suli’ (*Pyrus bretschneideri* Rehd. ‘Suli’) and ‘Huangli’ (*Pyrus pyrifolia* ‘Huangli’) were selected as parent varieties to investigate variations in the nutritional quality of the JBP at different developmental stages and to understand the process by which fragrance quality is formed after pollination. ‘Yali’ is often used for pollination due to its unique fragrance, delicate aroma, sweetness and crispness [3]. ‘Suli’ is often used for pollination due to its strong aroma and abundant edible fruits [10]. ‘Huangli’ is often used for pollination due to its status as an excellent local cultivar originally from the mountainous regions of Guizhou, China [11]. The aim of this research was to analyze the varying impact of xenia on the volatile compounds of the JBP and the connection between the nonvolatile metabolites and the development of aroma quality. By meticulously examining the intricacies of pollination, this endeavor establishes a robust and coherent theoretical foundation. It facilitates the determination of optimal pollination varieties for the JBP. It also deepens our understanding of xenia’s multifaceted impacts, leading to more comprehensive and interconnected research in this field.

## 2. Materials and Methods

### 2.1. Chemical and Reagents

The volatile compound standards for 33 aldehydes mixed standard in acetone (1000 μg/mL), 41 ketones mixed standard in acetone (1000 μg/mL) and 65 esters mixed standard in acetone (1000 μg/mL) were purchased from Alta Scientific (Tianjin, China). Formic acid and acetonitrile were purchased from Yuanye Biological Technology Co., Ltd. (Shanghai, China). Other chemical reagents were all domestically produced analytical pure and purchased from Aladdin (Shanghai, China).

### 2.2. Experimental Design

The experiment was carried out from 2023 in Mengwu Ecological Park, Mentougou District, Beijing, China. Three distinct types (‘Yali’, ‘Suli’, ‘Huangli’) of pollen were chosen to pollinate the JBP in three separate orchards. The fruits of ‘Jingbaili’ pear × ‘Yali’ pear (pollinator), ‘Jingbaili’ pear × ‘Suli’ pear (pollinator) and ‘Jingbaili’ pear × ‘Huangli’ pear (pollinator) were named the JY group, JS group and JH group, respectively. Six trees that were approximately 50 years old were selected for pollination in each orchard [12]. Each orchard was spaced around 10 m apart. Windless conditions were preferred to minimize any external disturbances. Additionally, the pollinated pears were covered with bags for a duration of 7 days after the pollination process [3]. The same cultivation management measures were applied to all three orchards. JBPs were collected randomly from different directions of each tree at the same height, with 10–20 pears collected each time [13]. In the bloom period after 50 d (24 May), sampling was conducted once every 30 days a total of four times, ending when the pears ripened on 4 September [13]. After picking, the pears were immediately transported to the laboratory. They were stored at 4 °C; then, a portion was taken for quality analysis. The remaining pears were ground into powder using liquid nitrogen and stored in a −80 °C refrigerator.

### 2.3. Measurement of Physical and Chemical Properties

The weight of each individual pear was measured with an electronic balance, while the lengthwise and widthwise dimensions of the pear were measured with vernier calipers (Figure S1). The phenolics extraction procedure involved measuring 0.20 g of a finely ground lyophilized sample. Then, 5 mL of an 80% methanol solution was added to the sample. The mixture was subjected to ultrasound-assisted extraction at room temperature for 30 min. Subsequently, the supernatant was separated using centrifugation (10,000 rpm, 10 min, 4 °C). This extraction process was repeated twice. Finally, the volume of the resulting solution was adjusted to 10 mL [14]. The extracts were used to determine the total phenolic, total flavonoid content and antioxidant capacity.

The Foline-Ciocalteu colorimetric method was used to determine the total phenolic content. Specifically, 0.2 mL of the extract was pipetted, and 5 mL of distilled water and 0.6 mL of 1 mol/L Foline-Ciocalteu reagent were added. The mixture was then mixed and allowed to stand for 5 min. After that, 1.0 mL of 15% Na_2_CO_3_ was added, and the volume was brought up to 10 mL with distilled water. The absorbance was measured at 760 nm after allowing the mixture to stand for 60 min at room temperature in the dark. A standard curve was obtained using gallic acid as a reference. The results were expressed as mg gallic acid equivalents (GAE)/g dry weight (DW), and each sample was assayed in parallel three times [15].

To determine the total flavonoids with the Aluminum Nitrate colorimetric method, 0.6 mL of the extract and 0.5 mL of 5% NaNO_3_ were pipetted into a 10 mL centrifuge tube. The mixture was then mixed and allowed to react at room temperature for 6 min. Subsequently, 0.5 mL of Al (NO_3_)_3_ was added to each tube, which was shaken well and allowed to react at room temperature for another 6 min. Then, 4 mL of NaOH was added, the volume was adjusted with water, and the mixture was shaken well again and allowed to stand at room temperature for 15 min. The absorbance was measured at a wavelength of 500 nm. A standard curve was obtained using rutin as a reference. The results were expressed as mg rutin equivalents (RE)/g DW, and each sample was assayed in parallel three times [15].

The antioxidant capacity was determined using DPPH, ABTS and FRAP kits from Sangon Biotech (Shanghai, China). We performed the assay according to the instructions provided with the kit. The soluble sugar content was determined using the anthrone colorimetric method [16], and the titratable acid content was determined through NaOH titration [17].

### 2.4. Volatile Compound Analysis

#### 2.4.1. Sample Preparation

Volatile compounds were extracted by a 50/30 μm divinylbenzene/carboxen/polydimethylsiloxane solid-phase microextraction (SPME) fiber (Supelco, Bellefonte, PA, USA) based on the literature [18]. Chopped ripe pears that had been lyophilized by liquid nitrogen grinding at room temperature were used for sample preparation. First, 1 g of the frozen sample and 6 mL of saturated NaCl solution (0.36 g/mL) were transferred into 20 mL SPME vials, and the vials were sealed immediately. The injection vial was stirred at 40 °C and equilibrated in solution and headspace after 20 min; then, the fibers were exposed to the headspace of the SPME vial for 20 min, and finally the fibers were withdrawn and inserted into the heated syringe port of the GC and resolved at 250 °C for 5 min in splitless mode.

#### 2.4.2. Gas Chromatographic Mass Spectrometry (GC-MS) Conditions

GC-MS analysis was performed by a gas chromatograph (TRACE 1600, Thermo Fisher Scientific, Waltham, MA, USA) coupled to a mass spectrometer (ISQ 7610, Thermo Fisher Scientific). Compound separation was performed on a TG-5MS (30 m × 0.25 mm × 0.25 μm, Thermo Scientific) column. Helium at a constant flow rate (1 mL/min) was used as the carrier gas. The GC was warmed up using the following procedure: the initial temperature was 30 °C and was held for 3 min; then, it was warmed up to 100 °C at 3 °C/min and held for 1 min; then, it was warmed up to 180 °C at 5 °C/min and held for 1 min; and finally it was warmed up to 280 °C at 10 °C/min and held for 5 min [19]. The temperature of the transfer line was 280 °C. The temperature of the ion source was set at 280 °C, and the mass spectrum was recorded at 70 eV in EI ionization mode with a scanning range of 50–500 amu.

The initial identification of compounds was done by comparing the mass spectra of the samples with the National Institute of Standards and Technology (NIST) database (https://www.sisweb.com/, accessed on 20 March 2024). Volatile compounds were determined with a match higher than 750. Mass spectrometry (MS) identification was confirmed by standards. The standards used in this research were diluted to a certain concentration with acetone, step by step (20 μg/mL, 10 μg/mL, 5 μg/mL, 2 μg/mL, 1 μg/mL), and the injection method was the same as that of the samples. Quantitative analysis was performed by plotting standard curves with the standards, data acquisition with Full Scan and selected ion monitoring (SIM) method.

### 2.5. Nonvolatile Metabolite Analysis

#### 2.5.1. Sample Preparation

For the sample preparation, 0.3 g of the sample was placed into a 10 mL centrifuge tube, and 5 mL of the methanol-acetonitrile water mixture (2:2:1) was added. The sample was then subjected to ultrasonicate for 60 min at 50 °C. Centrifugation was carried out at 10,000 rpm for 10 min at 4 °C, and the resulting supernatant was extracted with a small column of solid-phase extraction (Waters Oasis HLB). The treatment method is shown in Table S2. The eluent was nitrogen-blasted to dryness and then dissolved with 0.4 mL of methanol, after which it was passed through a 0.22 μm organic filtration membrane before liquid chromatography mass spectrometry (LC-MS) analysis [20]. Subsequently, Quality Control (QC) samples were created by combining 100 μL of each sample in an injection vial.

#### 2.5.2. LC-MS Conditions

The extract of the JBP was analyzed using a Vanquish HPLC system equipped with Orbitrap Exploris (Thermo Fisher Scientific, USA). The column temperature was 40 °C, the injection volume was 5 μL, the flow rate was 0.4 mL/min, the mobile phase A was 0.1% formic acid aqueous solution and the mobile phase B was 0.1% formic acid acetonitrile solution. The gradient elution was performed using the following program: 0 min, 5% B; 0.5 min, 5% B; 2.5 min, 40% B; 5.5 min, 50% B; 7.5 min, 100% B; 12.5 min, 100% B; 12.6 min, 5% B; and 17 min, 5% B [21].

The mass spectrum parameters are as follows: Full Scan ranges (*m*/*z*): 75–1000; Resolution: 60,000; RF Lens (%): 70; Intensity Threshold: 2 × 10^4^; Ion Source Type: H-ESI; Ion Transfer Tube Temp: 325 °C; Vaporizer Temp: 350 °C. The data were collected in the DDA mode.

The Compound Discoverer 3.3 program automatically identified these distinct metabolites from the raw data. The reliability of these metabolites should be confirmed by manual inspection. The structure of metabolites was identified using MS data search through the ChemSpider database and MS/MS spectrum mapping through the mzCloud database. Mass spectrometry data from 12 batches (Table S1) of the samples collected from UPLC-MS/MS were imported into the online analysis tool MetaboAnalyst 6.0 for multivariate statistical analysis.

### 2.6. Statistical Analysis

All data were generated from six experiments and exported to Microsoft Excel to be presented as the mean ± standard deviation (SD). Principal Component Analysis (PCA) and Partial Least Squares Discriminant Analysis (PLS-DA) with principal component loading plots and Hierarchical Cluster Analysis (HCA) with cluster heat maps were performed using MetaboAnalyst 6.0 (https://www.metaboanalyst.ca/, accessed on 25 July 2024). SPSS Statistics 17 (Armonk, New York, NY, USA) was used for significance analysis, and Graphpad Prism (version 8.0) was used to plot pie charts and bar graphs.

## 3. Results and Discussion

### 3.1. Effects of Xenia on the Physicochemical Properties of JBP

**Figure 1:**
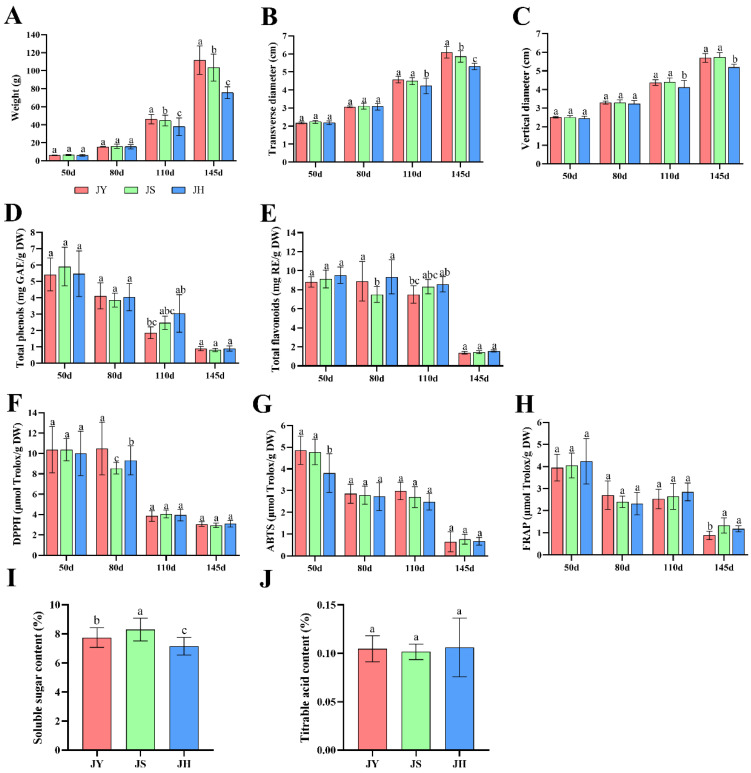
Effects of xenia on the physicochemical properties of JBP. (**A**) weight, (**B**) transverse diameter, (**C**) longitudinal diameter, (**D**) total phenol, (**E**) total flavonoid, (**F**) DPPH, (**G**) ABTS, (**H**) FRAP, (**I**) soluble sugar content, (**J**) titratable acid content. Bar graphs and error bars represent the mean and standard deviation. Different letters a, b and c indicate significant differences between samples (*p* < 0.05). JY: ‘Jingbaili’ pear × ‘Yali’ pear, JS: ‘Jingbaili’ pear × ‘Suli’ pear, JH: ‘Jingbaili’ pear × ‘Huangli’ pear. 50 d: 50 d after full bloom, 80 d: 80 d after full bloom, 110 d: 110 d after full bloom, 145 d: 145 d after full bloom.

The growth and development patterns of the JBP from different pollinations are essentially identical. Growth is comparatively slow during pre-fruit development, then transitions from rapid growth during the middle stage to a slowdown in the late stage. This indicates the presence of a significant xenia effect. At maturity, substantial differences were observed in the weight of a single fruit and the transverse and longitudinal diameters between the JH group, JY group and JS group (Figure 1A–C). Research conducted to identify appropriate pollinating varieties for the tropics found that pollen from parents with varying affinities could have a substantial impact on fruit weight, fruit longitudinal diameter and fruit diameter [22]. In research on the xenia effect in pomegranate, a substantial impact of pollen source on fruit weight, fruit longitudinal diameter and fruit diameter was observed [23].

Phenolics are the most important secondary metabolites in pears, with good scavenging ability for excess free radicals in the human body and good antioxidant activity. The levels of total phenol and total flavonoids in the JBP decreased gradually over time, reaching their peak at 50 days after blooming and their lowest point at maturity (Figure 1D,E). The antioxidant capacity of also showed a decreasing trend. The fruit exhibited the highest antioxidant capacity 50 days after flowering and the lowest at the ripening stage (Figure 1F–H). The xenia effect had an impact on the total phenolics, total flavonoids content and antioxidant capacity of the pears, but it was not significant. In research on the xenia effect in the Gala apple, it was found that different pollens had significant effects on fruit sugar-to-acid ratio, total phenolics and total flavonoids, while there was no significant effect on titratable acid [24]. It was also found that xenia had a significant effect on the total phenolics and total flavonoids content of kiwifruit [25].

Sugar and acid are the main components of pear flavor, and their content directly affects the taste and quality of the fruit. The xenia effect significantly impacted the fruit’s soluble sugar levels, but had no significant influence on its titratable acid levels (Figure 1I,J). Pollination from different varieties had significant effects on the soluble sugar content and titratable acid content of plum fruits, showing an obvious xenia effect [26]. It was found that the xenia effect improved the quality of blueberry fruits and had significant effects on soluble sugar content and titratable acid content [27].

Summarizing the findings, the JBP exhibited a clear xenia phenomenon in terms of single fruit weight, transverse and longitudinal diameters, and soluble sugar content. However, there was no apparent effect on total phenol, total flavonoid content, antioxidant capacity, or titratable acid content. Through a comprehensive analysis of the physicochemical properties of pears resulting from three different pollination combinations, it was found that pollination with the ‘Yali’ pear and ‘Suli’ pear is more suitable for JBP trees.

### 3.2. Effect of Xenia on Volatile Components of JBP

**Figure 2:**
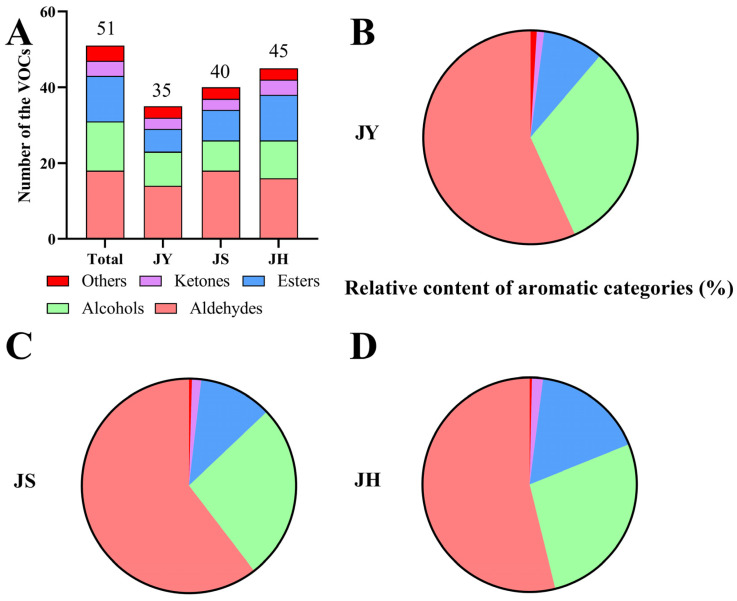
Effects of xenia on volatile compounds of ripe JBP. (**A**) Quantity of volatile compounds. (**B**) Changes in the relative content of volatile compounds in the pollination of ‘Yali’ pears. (**C**) Changes in the relative content of volatile compounds in the pollination of ‘Suli’ pears. (**D**) Changes in the relative content of volatile compounds in the pollination of ‘Huangli’ pears.

Flavor is an important indicator of fruit quality, and the flavor of pears directly affects their market acceptance and consumer satisfaction. The representative total ion chromatograms (TIC) of the JBP are presented in Figure S2. A total of 51 volatile compounds were detected, which can be categorized into 18 aldehydes, 12 esters, 13 alcohols, 4 ketones and 4 other compounds (Table S4). At maturity, the JY group contained 35 volatile compounds, consisting of 14 aldehydes, 6 esters, 9 alcohols, 3 ketones and 3 other compounds (Figure 2A). The fruits of the three differently pollinated JBPs contained a total of 29 volatile chemicals, primarily E-2-hexenal, 2-hexenal, (E, E)-2,4-hexadienal, 3-hexen-1-ol, 1-hexanol and hexyl acetate (Figure S3).

Aldehydes exhibit significant diversity among all the volatile compounds, followed by alcohol species and esters. Ketones and other compounds have a smaller range of types compared to aldehydes. Furthermore, the scent attributes of the pears are intricately linked not only to the quantities but also to the concentrations of volatile compounds. The proportion of aldehydes was notably high among the volatile compounds, with percentages of 56.82% (JY), 60.38% (JS) and 53.85% (JH), respectively (Figure 2B–D). Alcohols, which are the second most common aroma volatile, also exhibited variation across the pollination pears. Esters constituted the third most significant aroma component. Among the total volatile components, the level of other unclassified components was relatively low.

Aroma is an intricate blend of several volatile molecules, constituting merely a fraction of the mass of fresh fruit [28], yet it plays a significant role in determining fruit flavor. The decline in fruit flavor quality can be attributed to the reduction of aroma volatiles [7]. Prior research involved the identification of fragrance compounds in fully matured fruits of 202 pear varieties using HS-SPME-GC-MS. A total of 221 volatile components were discovered. The research revealed that aldehydes, esters and alcohols were the prevailing scent constituents, with aldehydes being the most prevalent molecules [29]. The volatile compounds of Korla balsam pears from 12 orchards were analyzed by HS-SPME-GC-MS, and a total of 100 volatile compounds were detected. Aldehydes were also found to be the most abundant compounds among the volatile compounds [30], which was in agreement with the results of our research.

**Figure 3:**
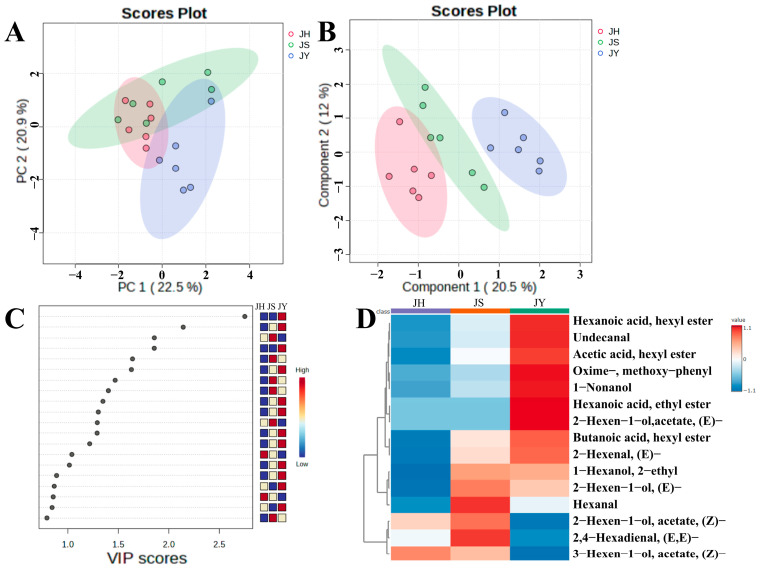
Effects of xenia on multivariate analysis of volatile compounds in ripe JBP. (**A**) PCA; (**B**) PLS-DA; (**C**) VIP > 1 in PLS-DA; (**D**) Clustering heat map analysis of differential metabolites (VIP > 1). The higher and lower relative contents are presented in red and blue, respectively. (For interpre-tation of the references to color in this figure legend, the reader is referred to the web version of this article).

PCA could not differentiate the volatile components in the JY, JS and JH groups, but there was a trend towards segregation, likely due to biological differences among the pear trees. The eigenvalues PC1 and PC2 were 22.5% and 20.9%, respectively (Figure 3A), with PC3, PC4 and PC5 accounting for 11.2%, 7.8% and 6.2%, respectively, summing to 68.6%. Supervised PLS-DA reduced within-group variation and maximized between-group variation, allowing for better characterization of the variability across the samples and aiding in the search for differential metabolites [31]. The results of the PLS-DA showed that there was a significant difference between the three differently pollinated JBPs (Figure 3B). The R^2^ and Q^2^ of the five-fold cross-test results of the PLS-DA were 0.9330 and 0.7530, respectively (Figure S4), indicating that the PLS-DA is reliable and is not overfitted.

In order to characterize the variability of volatile compounds in the three differently pollinated JBPs, we used a PLS-DA model to screen 15 differentially volatile compounds with VIP > 1 (Figure 3C). These compounds included Z-2-hexen-1-ol acetate, hexyl butyrate, E-2-hexen-1-ol acetate, ethyl caproate, oxime-methoxy-phenyl-, hexyl acetate and so on. A clustered heat map was used to depict the relative abundance of these 15 compounds (Figure 3D). The heat map clearly showed significant variations in their concentrations among the three differently pollinated JBPs. Specifically, E-2-hexen-1-ol acetate concentrations were higher in the JH group, while hexanal and (E, E)-2,4-hexadienal were higher in the JS group. Additionally, hexyl acetate, oxime-methoxy-phenyl- and Z-2-hexen-1-ol acetate were relatively more abundant in the JY group. The xenia effect had significant impact on the composition and content of volatiles in the JBPs, showing that different pollens had significant effects on volatile compounds.

Hexyl acetate, E-2-Hexenal and (E, E)-2,4-Hexadaienal were identified in all the JBP samples, with high contents. These compounds may be characteristic of the JBP and contribute to its aroma. Hexyl acetate possesses a rich, fruity aroma, E-2-hexenal exhibits a strong grassy aroma and (E, E)-2,4-hexadienal features a unique floral-fruity aroma and is the main contributor to the fruity, grassy and floral odors of the JBP. In order to determine the specific content of volatile components in the JBPs from three different pollinations, a total of 13 representative volatile compounds were selected for quantitative research. Table 1 and Table S3 display the standards, standard curves and content values for each compound. Additionally, standard equations for the 13 compounds were constructed to determine the content of volatile components in the JBP using peak area calculations. The correlation coefficients (R^2^) for all standard curves were ≥0.9900, indicating a strong linear relationship for each substance’s standard curve.

The main volatile compounds found in the three differently pollinated JBPs were 1-nonanol, E-2-hexen-1-ol, hexanal, E-2-hexenal, hexyl acetate, ethyl hexanoate and hexyl hexanoate. The production of these volatile components in fruits primarily occurs through the plant secondary metabolism, involving various biochemical metabolic pathways [32]. The lipid-derived fatty acid synthesis process and the protein-derived amino acid synthesis pathway are the primary sources of straight- and branched-chain alcohols, aldehydes, esters and other chemicals that contribute to the scent of fruits [33]. The main mechanism of fatty acid synthesis is the lipoxygenase (LOX) pathway [34]. Previous studies have consistently shown a strong correlation between LOX activity and the presence of aldehydes and esters [35].

Volatile compounds in fruits, such as alcohols, aldehydes, esters and other low-carbon compounds, are the main source of floral, fruity and ester aroma components. Numerous compounds are produced through anabolic pathways, where amino acids serve as precursor substances [36]. Previous studies have found that isoleucine may be precursors of volatile alcohols, aldehydes and esters in apples [37]. Research has also shown that flavor formation in strawberries is related to amino acid metabolism [38]. The differential volatile compounds identified in this research, such as hexyl acetate, hexyl butyrate, ethyl caproate and hexyl caproate, were primarily derived from the amino acid synthesis pathway. Therefore, the amino acid metabolic pathway is potentially the key to explaining the variations in aroma characteristics among the differently pollinated JBPs.

### 3.3. Effects of Xenia on Nonvolatile Metabolites of JBP

The composition and relative amounts of metabolites, including sugars, organic acids, polyphenols and amino acids, have a crucial role in defining the quality and flavor of fruits [39]. Hence, examining the influence of xenia on metabolites is of paramount importance. The chromatographic results of the QC samples in positive and negative ion modes are displayed in Figure S5. The results showed that the peak areas and retention times of all the QC samples are in close agreement. A total of 229 metabolites were identified by combining the positive and negative ion chromatograms. These metabolites include seven sugars (such as L-Iditol, sucrose, D-mannose and D-lactose monohydrate), 41 organic acids (such as D-Quinic acid, D-Malic acid, and Fumaric acid), 23 polyphenols (such as Chlorogenic acid, Catechin and rutin), 22 amino acids (such as D-Tryptophan, Asparagine, Proline and Valine) and their derivatives, three nucleotides and their derivatives, four vitamins (such as Nicotinamide) and other secondary metabolites (Table S5).

**Figure 4:**
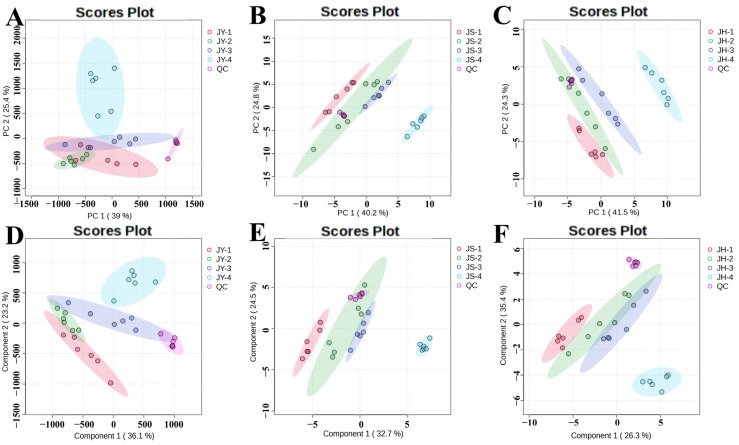
Effects of different developmental stages on multivariate analysis of nonvolatile metabolites in JBP. PCA (**A**) and PLS-DA (**D**) of metabolites of JY group; PCA (**B**) and PLS-DA (**E**) of metabolites of JS group; PCA (**C**) and PLS-DA (**F**) of metabolites of JH group.

To conduct a more detailed analysis of the impact of various developmental phases on the metabolites of the JBP, samples were taken from four distinct developing stages of each pollinated JBP and evaluated individually. Figure 4 demonstrates that the four developmental phases in the PCA model for the JY group were not easily distinguishable and exhibited some overlap, whereas the four developmental stages in the PCA models for the JS group and the JH group were clearly distinct from each other. Some samples exhibited greater dispersion, possibly due to the heterogeneity introduced by their biological replicates, but these inherent variances did not impact the differentiation effect.

Following unsupervised PCA modeling, a supervised PLS-DA analysis was conducted on the mass spectrometry data mentioned above. Figure 4 clearly displays the four developmental stages of the three groups of JBP with varied pollination methods. The separation of the samples is more pronounced, and the degree of separation is enhanced compared to the PCA model. The R^2^ and Q^2^ values obtained from the five-fold cross-test were 0.9877 and 0.9170, 0.9986 and 0.7830, and 0.9928 and 0.8518, respectively (Figure S6). These values indicate that the PLS-DA model was both reliable and stable, and it effectively explained the data without overfitting. The PLS-DA analysis revealed significant variations among the four developmental stages within each group.

**Figure 5:**
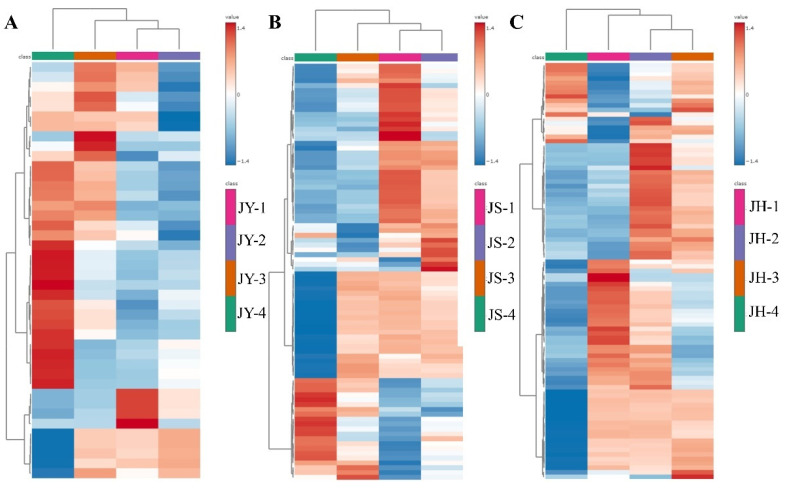
Clustered heat map analysis of differential metabolites at different developmental stages of each pollinated JBP. (**A**) JY group, (**B**) JS group, (**C**) JH group.

In order to identify differences between the developmental stages of the three differently pollinated JBPs, the VIP values of the PLS-DA model were used to screen the differential compounds. A total of 37 (JY), 81 (JS) and 91 (JH) differential metabolites with a VIP > 1 were identified using the PLS-DA model. These compounds accounted for the significant variations observed between the different developmental stages of the JBP. In order to visually represent the relative content levels of these differential metabolites, we analyzed the compounds using a heat map based on mass spectrometry signal intensity (Figure 5). The heat map clearly shows significant differences in the four developmental stages of the JBPs with different pollination.

Fruit development is a complex process involving multiple stages and influencing factors. In order to clarify the metabolic changes during fruit development, PCA and PLS-DA were used to analyze the JBP at different developmental stages. The results revealed significant differences in metabolite compositions across the stages, with the most prominent changes occurring during ripening. By screening differential metabolites by VIP > 1 in PLS-DA and analyzing them by drawing heat maps with differential metabolites, it can be found that the compounds with relatively high content in the ripening stage of the three differently pollinated JBPs are sucrose, D-mannose, D-hydroxy lactose, citric acid and other substances. Sucrose accumulates gradually with fruit development, and its content increases dramatically during ripening. Starch and organic acids are converted to sugar, which is consumed to a relatively small degree, and sugar accumulates [40]. Citric acid increased significantly at maturity, a result that was similar to others’ studies [41]. In light of the preceding results, it was found that the JBPs with different pollinations differed significantly at maturity, and further investigation was desired to explore the metabolic changes that occurred during the development of each of these pears subjected to different pollinations.

**Figure 6:**
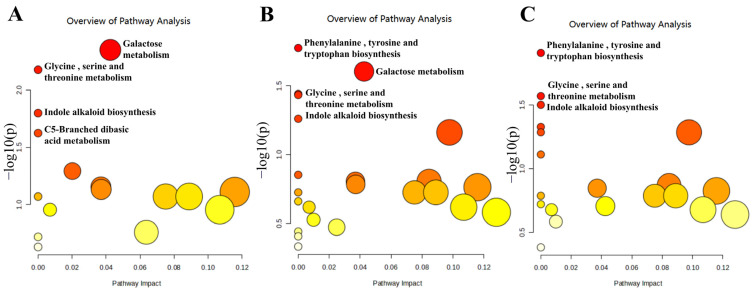
Metabolic pathway enrichment analysis of differential metabolites at different developmental stages of each pollinated JBP fruit. (**A**) JY group, (**B**) JS group, (**C**) JH group. (For interpretation of the references to color in this figure legend, the reader is referred to the web version of this article).

Based on the KEGG database, pathway enrichment analysis of differential metabolites was performed by the online analysis tool MetaboAnalyst 6.0. As shown in Figure 6, among the 16 pathways enriched in the JY group, galactose metabolism, glycine, serine and threonine metabolism, indole alkaloid biosynthesis and the C5-branched dicarboxylic acid metabolism pathways were significantly enriched (*p* < 0.05). Among the 23 metabolism pathways enriched in the JS group, phenylalanine, tyrosine and tryptophan biosynthesis, galactose metabolism, glycine, serine and threonine metabolism, and the indole alkaloid biosynthesis pathway were significantly enriched (*p* < 0.05). Among the 20 metabolic pathways enriched in the JH group, phenylalanine, tyrosine and tryptophan biosynthesis, glycine, serine and threonine metabolism, and the indole alkaloid biosynthesis pathway were significantly enriched (*p* < 0.05).

Sugar is an essential nutrient for plant metabolism and regulates the growth process by participating in sugar metabolism [42]. The galactose metabolism exhibited considerable enrichment among differential metabolites, potentially exerting a crucial influence on fruit ripening in the JY and JS groups but not in the JH group. This disparity in the galactose metabolism may explain the variations observed in different pollination scenarios. Similar conclusions have been reported in other studies, such as those analyzing the composition of differently pollinated kiwifruit at different developmental stages by metabolomics, which identified the galactose metabolic pathway as the main pathway [43]. Amino acids play a crucial role in the process of fruit ripening and development. Analysis of the KEGG pathway enrichment revealed significant differences in the phenylalanine metabolic pathway between the JS and JH groups. Thus, the phenylalanine metabolic pathway may be pivotal in determining pear quality, and it may be responsible for the differences in ripe fruit quality among the three pollinators.

The research revealed significant differences in ripening among the differently pollinated JBPs, prompting further investigation into the metabolic changes during their growth. PCA of four distinct developmental stages of the three differently pollinated JBP revealed that there was partial overlap in the first stage, a tendency to separate in the second stage and clear separation in the third and fourth stages. These findings suggested that there are variations in metabolites among the differently pollinated JBPs.

**Figure 7:**
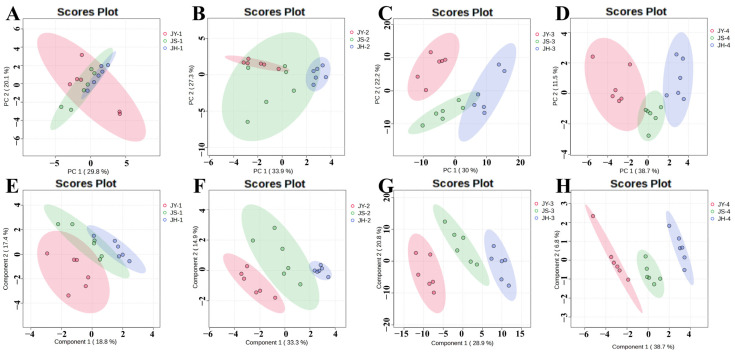
Effects of xenia on multivariate analysis of nonvolatile metabolites in JBP. PCA (**A**) and PLS-DA (**E**) of metabolites at the first developmental stages (50 days after flowering); PCA (**B**) and PLS-DA (**F**) of metabolites from the second developmental stage (80 days after flowering); PCA (**C**) and PLS-DA (**G**) of metabolites from the third developmental stage (110 days after flowering); and PCA (**D**) and PLS-DA (**H**) of metabolites from the fourth developmental stage of maturation (145 days after flowering).

Following unsupervised PCA modeling, a supervised PLS-DA analysis was conducted on the mass spectrometry data. Figure 7 illustrates the distinction between the three different pollination samples of the JBP. The separation of these samples was superior to that of the PCA model. The R^2^ and Q^2^ values obtained from the five-fold cross-validation tests are shown in Figure S7. The PLS-DA results showed that the three kinds of differently pollinated JBP differed significantly at each developmental stage and became larger as development progressed.

PCA and PLS-DA were used to stoichiometrically analyze the metabolites of the JBP. The findings revealed distinct differentiation among the JY, JS and JH groups, indicating that different pollens had an impact on the metabolites. The pollination of the ‘Dangshan Su’ pear with ‘Wonhwang’ pear and ‘Jingbaili’ pear pollen revealed that different pollens had a influence on metabolites, which was consistent with our results [12]. Similarly, the pollination of the ‘Yali’ pear with ‘Hongnanguo’ pear pollen and ‘Xuehuali’ pear pollen showed that metabolites and genes differed depending on the pollen used [3].

**Figure 8:**
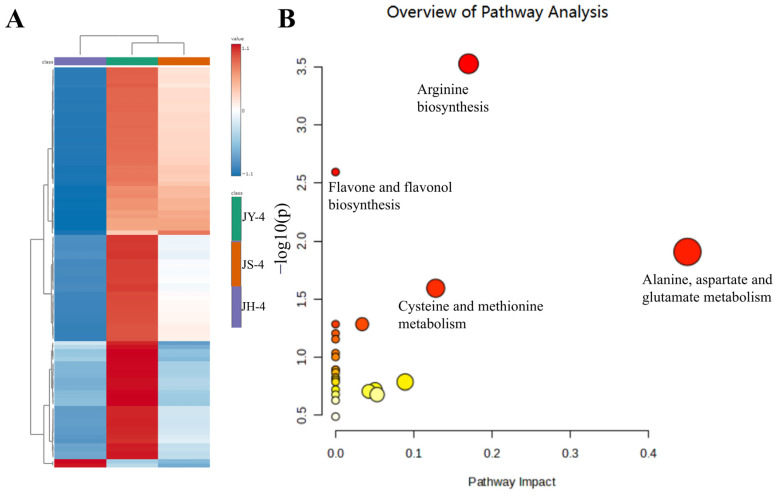
Effects of xenia on multivariate analysis of nonvolatile metabolites in ripening JBP. (**A**) Clustering heat map analysis, (**B**) enrichment analysis.

Since the differences were greatest at fruit maturity, only JBPs at the ripening stage were screened for differential compounds. Based on the PLS-DA, a total of 98 differential metabolites with VIP > 1 was identified, and these compounds exhibited significant differences among the three differently pollinated JBPs. In order to visually represent the relative content levels of these differential metabolites in the different JBPs, heat map analysis was carried out using the response intensities of their mass spectrometry signals (Figure 8A). The heat map clearly showed that the relative contents of differential metabolites in the three differently pollinated JBPs varied greatly. Specifically, the compounds with the highest relative contents in the JY group were lignans, jasmonic acid, L-aspartic acid and L-glutamic acid; the compound with the highest relative content in the JS group was sucrose; and the compound with the highest relative content in the JH group was rhizopodophyllin.

Based on the KEGG database, 98 differential metabolites were analyzed for pathway enrichment by the online analysis tool MetaboAnalyst 6.0 (Figure 8B). A total of 22 metabolic pathways were enriched for arginine biosynthesis, flavonoid and flavonol biosynthesis, alanine, aspartate and glutamate metabolism, cysteine and methionine metabolism, nitrogen metabolism, β-alanine metabolism, photosynthetic biocarbon fixation, starch and sucrose metabolism, galactose metabolism, glyoxylate and dicarboxylic acid metabolism. As shown in Figure 5, among the 22 pathways enriched to the ground, arginine biosynthesis, flavonoid and flavonol biosynthesis, alanine, aspartate and glutamate metabolism, and the cysteine and methionine metabolism pathways were significantly enriched (*p* < 0.05).

The KEGG enrichment analysis revealed variations in sugar metabolism, tricarboxylic acid (TCA) cycle, flavonoid biosynthesis, amino acid metabolism and other metabolites that could impact the flavor and nutrition of pears in differently pollinated JBPs. These differences may directly contribute to variations in flavor, nutrition and aroma among the differently pollinated JBPs. The pathways that showed significant enrichment were arginine biosynthesis, flavonoid and flavonol biosynthesis, alanine, aspartate and glutamate metabolism, and arginine and proline metabolism. In these pathways, arginine is mainly synthesized from aspartate and glycine, as well as from glutamate [44]. The primary metabolic processes for arginine include the creation of nitric oxide, urea and polyamines. The conversion of proline metabolism through its metabolism to pyruvate allows it to enter the tricarboxylic acid cycle [45]. Therefore, the differences in amino acid metabolism during the fruit development of the JBP may be the main reason for the differences in their ripening stages.

## 4. Conclusions

This research indicates significant differences in the physicochemical properties of three differently pollinated groups of the JBP. Compounds such as hexyl acetate, E-2-hexenal and (E, E)-2,4-hexadaienal may be aroma characteristic compounds in the JBP, contributing fruity, grassy and floral aromas to the JBP. The content of aroma-characteristic compounds in the JY group exceeded that in the JS group and JH group. The discovery of amino acid metabolism pathways may be the key to explaining the changes in the aroma characteristics of the differently pollinated JBPs. Furthermore, an examination of nonvolatile metabolites indicated that variations in galactose metabolism and phenylalanine metabolism could underlie the pollination disparities observed in the mature JBPs. The differences in amino acid metabolism during the development of the JBPs may be the main reason for the different pollination differences. The quality of the JBP can be enhanced by improving the pollinated varieties. This research can provide theoretical support for improving the quality of the JBP.

## Figures and Tables

**Table 1:** The effect of xenia on the volatile compounds content during the ripening of JBP.

Compounds	JY (μg/kg DW)	JS (μg/kg DW)	JH (μg/kg DW)
E-2-Hexenal	163.40 ± 201.61 ^a^	79.46 ± 19.15 ^b^	162.18 ± 253.26 ^a^
Heptanal	1.47 ± 0.26 ^a^	0.79 ± 0.16 ^b^	1.29 ± 0.19 ^a^
(E, E)-2,4-Hexadaienal	137.80 ± 217.29 ^b^	219.68 ± 159.07 ^a^	70.43 ± 106.88 ^c^
Octanal	5.11 ± 1.01 ^a^	3.04 ± 0.39 ^b^	3.17 ± 0.06 ^b^
Benzeneacetaldehyde	3.63 ± 2.93 ^a^	3.91 ± 2.83 ^a^	0.96 ± 0.81 ^b^
E-2-Octenal	2.24 ± 0.65 ^a^	2.14 ± 0.79 ^a^	1.82 ± 0.53 ^b^
Nonanal	12.69 ± 0.49 ^a^	7.72 ± 0.77 ^b^	7.58 ± 0.32 ^b^
Decanal	8.99 ± 2.71 ^a^	4.91 ± 0.40 ^b^	2.13 ± 0.73 ^c^
Dodecanal	6.67 ± 0.67 ^a^	6.51 ± 0.28 ^a^	6.54 ± 0.36 ^a^
Hexyl acetate	87.30 ± 34.22 ^a^	45.85 ± 29.63 ^b^	25.25 ± 17.73 ^c^
Hexyl caproate	5.28 ± 0.28 ^a^	5.28 ± 0.37 ^a^	5.03 ± 0.25 ^a^
1-Octen-3-one	4.18 ± 0.96 ^a^	1.47 ± 0.99 ^b^	0.66 ± 0.56 ^c^
5-Hepten-2-one, 6-methyl-	3.54 ± 0.34 ^a^	2.93 ± 0.99 ^b^	3.04 ± 0.16 ^b^
Total volatile compounds	442.30	383.69	290.08

Note: Different letters a, b and c indicate significant differences between samples (*p* < 0.05).

## Data Availability

The original contributions presented in the study are included in the article/Supplementary Materials, further inquiries can be directed to the corresponding author.

## Supplementary Materials

The following supporting information can be downloaded at: https://www.mdpi.com/article/10.3390/foods14010094/s1, Figure S1: The pear photos of self-collected ‘Jingbaili’ pear samples; Figure S2: TIC of the ‘Jingbaili’ pear samples, based on GC-MS (A: JY, B: JS, C: JH); Figure S3: Venn diagram of volatile compounds of the ‘Jingbaili’ pear samples; Figure S4: The five-fold cross-testing of the PLS-DA model in GC-MS analyze of ‘Jingbaili’ pear. R2 and Q2 represented the explanatory rate and predictive ability of the model, respectively, and the close their values were to 1 indicated that the model was more effective; Figure S5: TIC of QC samples in positive (A) and negative ions (B), based on UPLC-MS/MS; Figure S6: The five-fold cross-testing of PLS-DA model in UPLC-MS/MS analyze of each pollinated ‘Jingbaili’ pear at different developmental stages (A: JY, B: JS, C: JH); Figure S7: The five-fold cross-testing of the PLS-DA model in UPLC-MS/MS analyze of different pollinated ‘Jingbaili’ pear at four different developmental stages (A: 50 d, B: 80 d, C: 110 d, D: 145 d); Table S1: Collecting information of 12 batches of different pollinations ‘Jingbaili’ pear fruits; Table S2: Solid phase extraction (SPE) columns for sample treatment; Table S3: Standard curves and R2 for volatile compounds during the ripening of ‘Jingbaili’ pear by GC-MS; Table S4: Relative content of volatile compounds identified in different pollinated ‘Jingbaili’ pear by GC-MS; Table S5: Identification of compounds in ‘Jingbaili’ pear by UPLC-MS/MS.
