# Supplementary material for: Xenia Effect on Nutritional and Flavor Components of ‘Jingbaili’ Pear
Source: Foods. 2025 Jan 2;14(1):94. doi: 10.3390/foods14010094 (PMC11720288; doi:10.3390/foods14010094)
Supplement: Supplementary file 1 [file foods-14-00094-s001.zip › foods-3355473-supplementary.pdf]

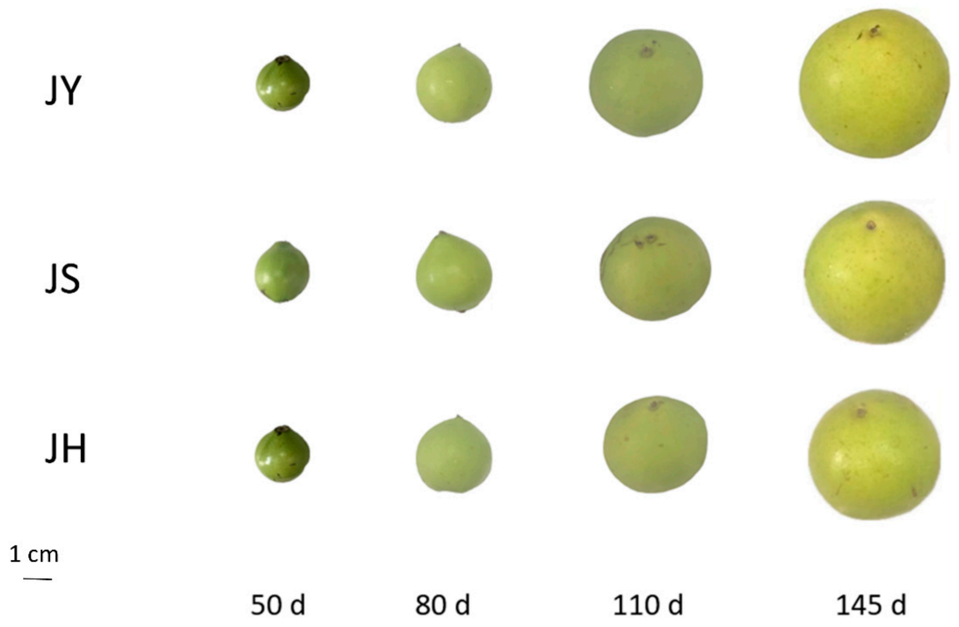

Figure S1. The pear photos of self-collected 'Jingbaili' pear samples.

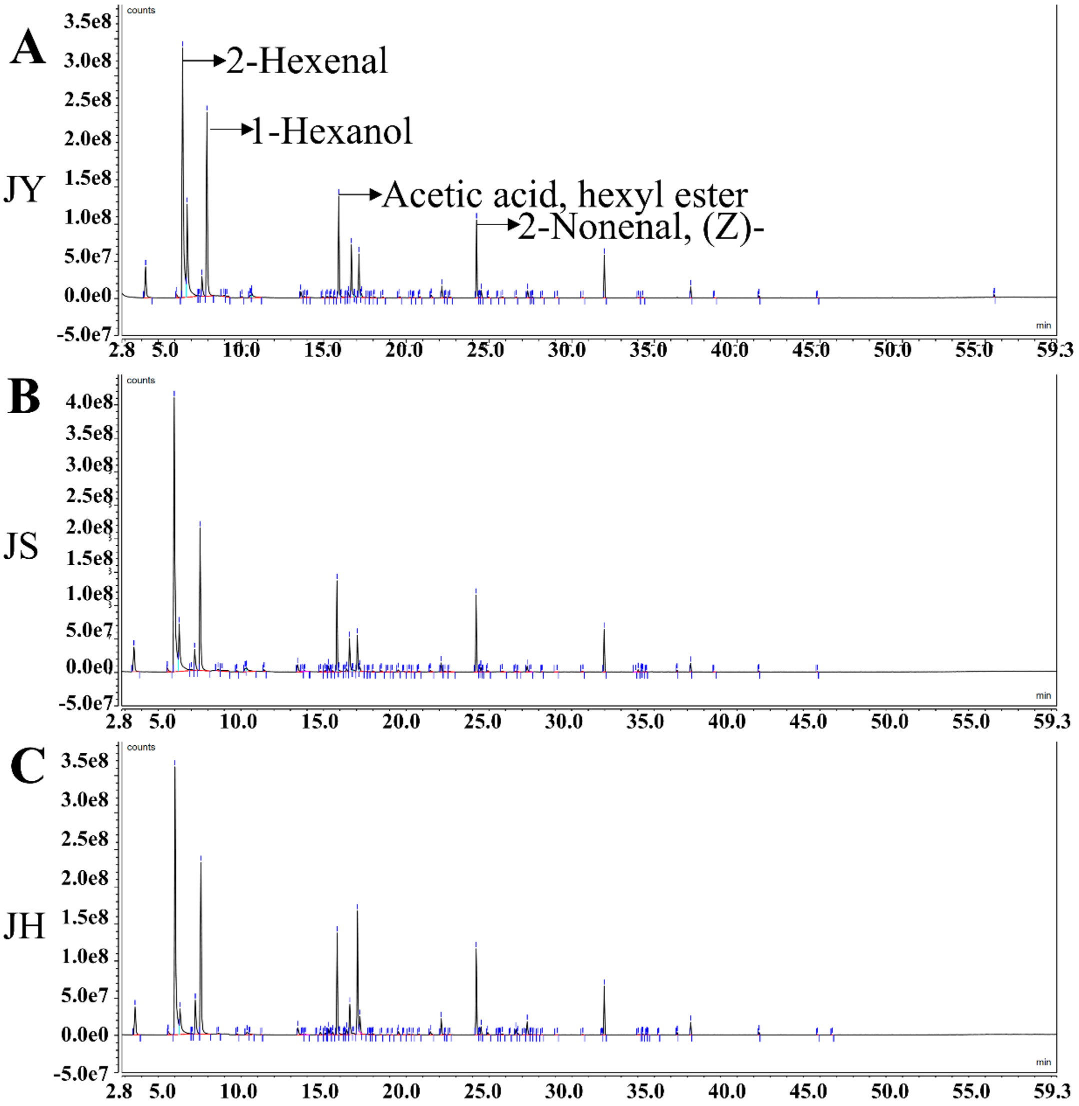

Figure S2. TIC of the 'Jingbaili' pear samples, based on GC-MS (A: JY, B: JS, C: JH).

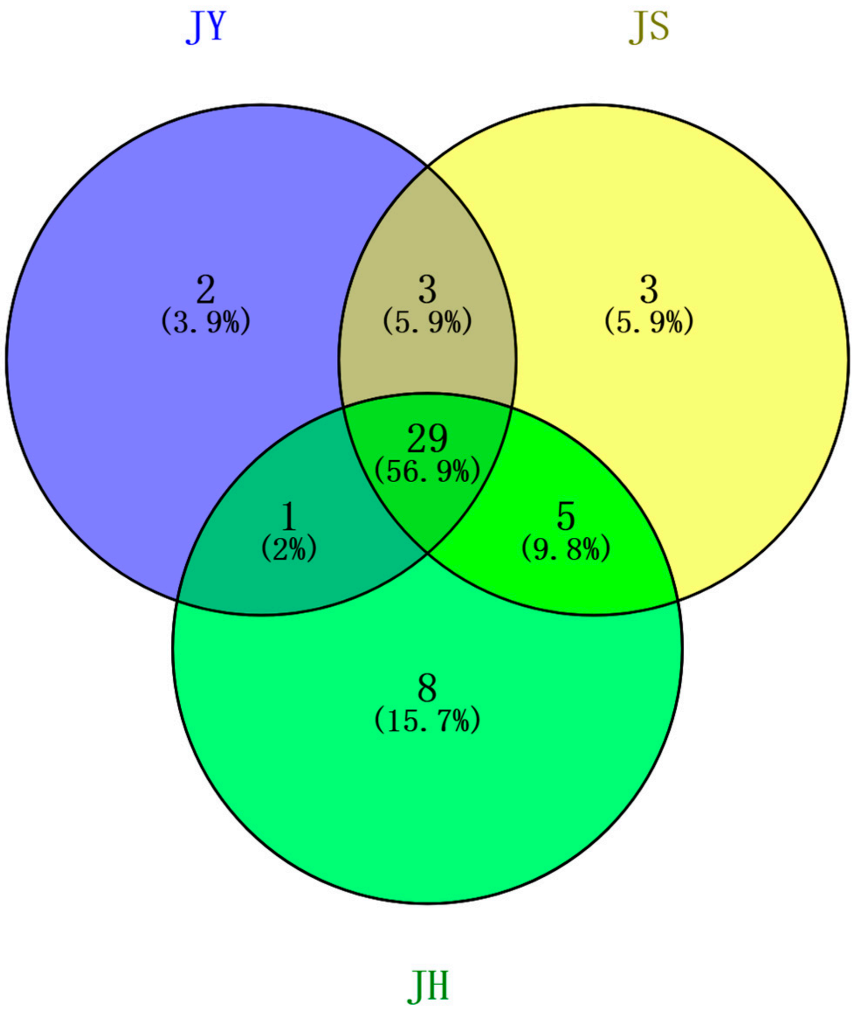

Figure S3. Venn diagram of volatile compounds of the 'Jingbaili' pear samples.

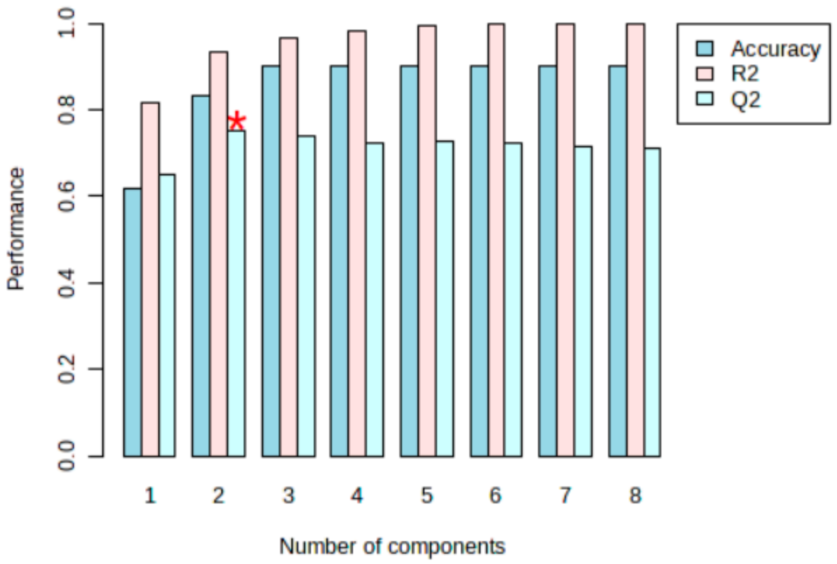

Figure S4. The five-fold cross-testing of the PLS-DA model in GC-MS analyze of 'Jingbaili' pear. R^2 and Q^2 represented the explanatory rate and predictive ability of the model, respectively, and the close their values were to 1 indicated that the model was more effective.

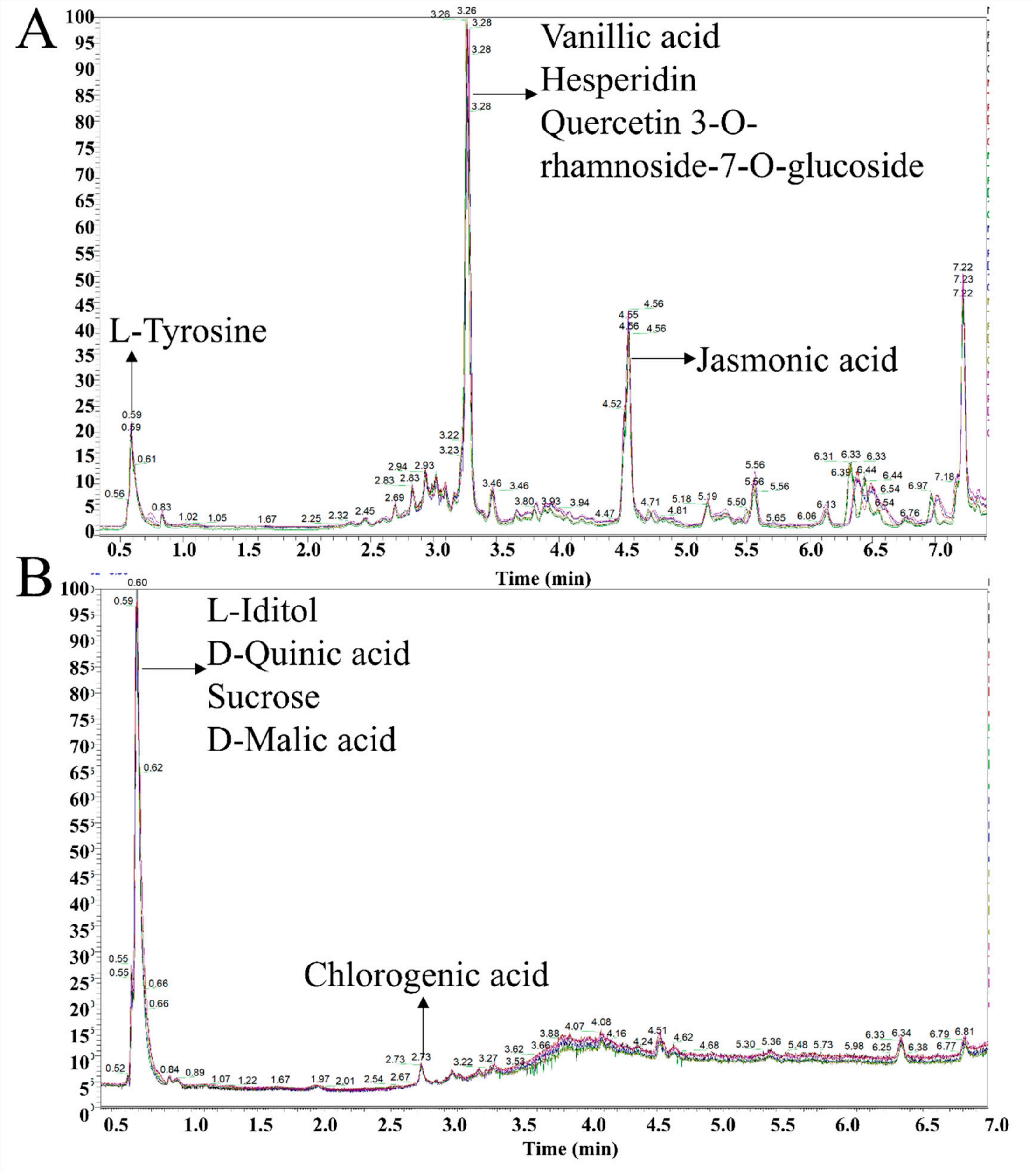

Figure S5. TIC of QC samples in positive (A) and negative ions (B), based on UPLC-MS/MS.

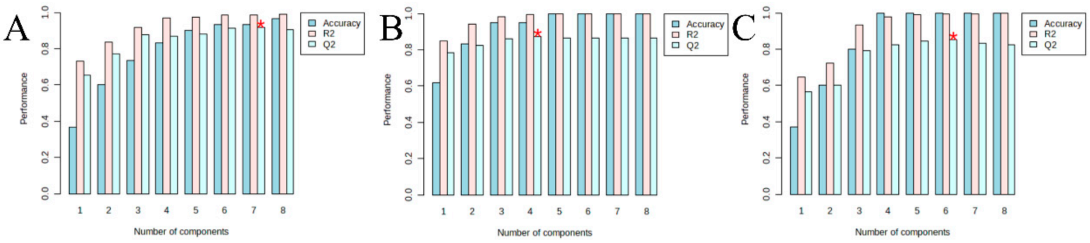

Figure S6. The five-fold cross-testing of PLS-DA model in UPLC-MS/MS analyze of each pollinated ‘Jingbaili’ pear at different developmental stages (A: JY, B: JS, C: JH).

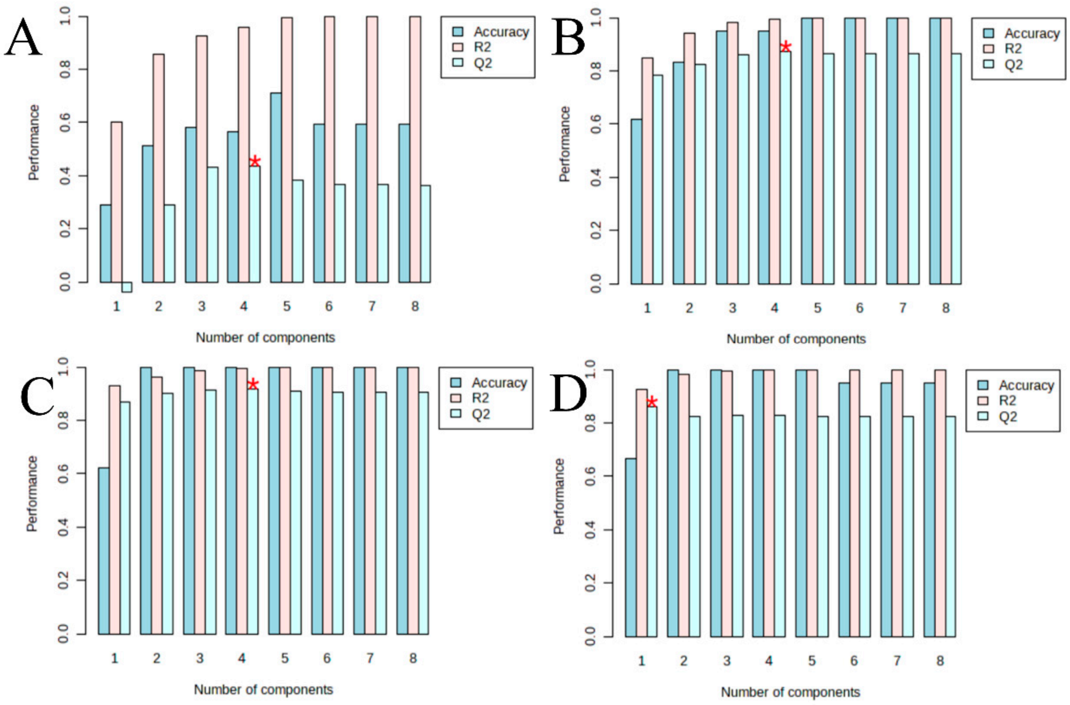

Figure S7. The five-fold cross-testing of the PLS-DA model in UPLC-MS/MS analyze of different pollinated ‘Jingbaili’ pear at four different developmental stages (A: 50 d, B: 80 d, C: 110 d, D: 145 d).

Table S1. Collecting information of 12 batches of different pollinations 'Jingbaili' pear fruits.

Sample	Pollinations	Harvest time	Sample size
S1	JY	50 d	18
S2	JS	50 d	18
S3	JH	50 d	18
S4	JY	80 d	12
S5	JS	80 d	12
S6	JH	80 d	12
S7	JY	110 d	12
S8	JS	110 d	12
S9	JH	110 d	12
S10	JY	145 d	6
S11	JS	145 d	6
S12	JH	145 d	6

Table S2. Solid phase extraction (SPE) columns for sample treatment.

	**Water Oasis HLB**
Activation	Methanol 5 mL
Equilibrium	Methanol acetonitrile water mixed solution 3 mL
Loading sample	Extraction fluid 3 mL
Sprinkle	Water 3 mL
Purge	Methanol 5 mL

Table S3. Standard curves and R^2 for volatile compounds during the ripening of 'Jingbaili' pear by GC-MS.

Compounds	Standard curve	R^2
E-2-Hexenal	y=25065x+38106	0.9934
Heptanal	y=111458x+141147	0.9929
(E, E)-2, 4-Hexadaienal	y=14860x-384.69	0.9979
Octanal	y=283806x+172950	0.9928
Benzeneacetaldehyde	y=52882x+24192	0.9990
E-2-Octenal	y=271476x+105724	0.9995
Nonanal	y=389587x+466536	0.9915
Decanal	y=418135x+528889	0.9909
Dodecanal	y=227154x+378603	0.9923
Hexyl acetate	y=323746x+592088	0.9920
Hexyl caproate	y=656243x+866643	0.9910
1-Octen-3-one	y=444047x+519219	0.9908
5-Hepten-2-one, 6-methyl-	y=300554x+728697	0.9907

Table S4. Relative content of volatile compounds identified in different pollinated 'Jingbaili' pear by GC-MS.

No.	Identification	CAS	RT (min)	Relative content (%)		
				JY	JS	JH
Aldehyde						
1	2-Hexenal, (E)-	6728-26-3	6.09	1.75±2.15	0.77±0.23	1.63±2.51
2	2-Hexenal	505-57-7	6.471	48.11±20.2	44.42±8.79	34.78±19.65
3	Heptanal	111-71-7	10.055	0.17±0.09	0.17±0.02	0.15±0.08
4	2, 4-Hexadienal, (E, E)-	142-83-6	10.562	0.85±1.33	1.17±0.84	0.4±0.61
5	Octanal	124-13-0	16.49	0.66±0.29	0.56±0.21	0.83±0.16
6	Benzeneacetaldehyde	122-78-1	18.572	0.12±0.15	0.11±0.13	0.04±0.09
7	2-Octenal, (E)-	2548-87-0	19.578	0.38±0.11	0.36±0.07	0.4±0.1
8	Nonanal	124-19-6	22.139	1.99±0.72	1.74±0.77	2.63±0.65
9	Decanal	112-31-2	27.326	1.24±0.4	1.49±0.82	2.27±0.38

10	2-Octenal, 2-butyl-	13019-16-4	34.012	0.12±0.1	0.11±0.11	0.08±0.06
11	2-Heptenal, (Z)-	57266-86-1	13.613	0.8±0.74	0.6±0.54	0.94±0.25
12	2-Nonenal, (Z)-	60784-31-8	24.935	0.35±0.29	0.46±0.21	0.54±0.33
13	2-Heptenal, (E)-	18829-55-5	11.381	0.16±0.4	0.13±0.31	0.00
14	3-Hexenal, (Z)-	6789-80-6	6.09	0.12±0.3	0.1±0.26	0.00
15	Undecanal	112-44-7	31.805	0.00	0.04±0.06	0.08±0.01
16	3-Hexenal	4440-65-7	2.967	0.00	0.09±0.22	0.00
17	Hexanal	66-25-1	3.063	0.00	8.06±17.82	9.08±15.77
18	Dodecanal	112-54-9	35.257	0.00	0.01±0.03	0.01±0.03
Alcohols						
1	3-Hexen-1-ol	544-12-7	6.743	10.05±7.59	6.81±3.78	4.02±3.27
2	2-Hexen-1-ol, (E)-	928-95-0	7.634	2.37±1.95	2.2±0.88	2.92±2.35
3	1-Hexanol	111-27-3	7.943	18.79±6.25	16.72±3.51	19±10.1

4	1-Hexanol, 2-ethyl-	104-76-7	18.058	0.11±0.12	0.17±0.06	0.08±0.09
5	1-Nonen-4-ol	35192-73-5	21.497	0.23±0.19	0.24±0.13	0.22±0.2
6	2-Hexyn-1-ol	764-60-3	6.056	0.16±0.38	0.00	0.1±0.24
7	3-Hexen-1-ol, (Z)-	928-96-1	5.688	0.19±0.46	0.00	0.00
8	2-Hexen-1-ol, (Z)-	928-94-9	3.365	0.07±0.16	0.32±0.78	0.00
9	1-Nonanol	143-08-8	25.632	0.01±0.03	0.01±0.03	0.04±0.05
10	Bicyclo[2.1.1]hexan-2-ol, 2-ethenyl-	511519-83-8	7.378	0.00	0.24±0.38	0.00
11	3-Hexen-1-ol, (E)-	928-96-1	5.688	0.00	0.00	0.78±1.9
12	1-Octen-3-ol	3391-86-4	14.976	0.00	0.00	0.05±0.08
13	4-Methyl-5-decanol	213547-15-0	21.408	0.00	0.00	0.07±0.17
Ester						

1	3-Hexen-1-ol, acetate, (Z)-	3681-71-8	16.667	3.75±2.6	1.08±1.85	0.74±1.17
2	Acetic acid, hexyl ester	142-92-7	17.133	4.35±3.35	6.15±4.28	11.81±5.19
3	2-Hexen-1-ol, acetate, (E)-	2497-18-9	17.286	0.23±0.27	0.41±0.34	0.12±0.31
4	3-Hexen-1-ol, acetate, (E)-	3681-82-1	16.664	0.51±1.25	2.98±2.54	2.19±1.81
5	Butanoic acid, hexyl ester	2639-63-6	26.632	0.04±0.07	0.17±0.09	0.44±0.16
6	2-Hexen-1-ol, acetate, (Z)-	56922-75-9	17.269	0.26±0.64	0.24±0.59	0.89±1.03
7	Hexanoic acid, ethyl ester	123-66-0	16.163	0.00	0.02±0.05	0.1±0.13

8	Butanoic acid, 2-hexenyl ester, (Z)-	56922-77-1	26.772	0.00	0.02±0.04	0.03±0.08
9	Butanoic acid, 2-hexenyl ester, (E)-	53398-83-7	26.737	0.00	0.00	0.13±0.11
10	Hexanoic acid, hexyl ester	6378-65-0	34.526	0.00	0.00	0.06±0.05
11	3-Hexen-1-ol, formate, (Z)-	33467-73-1	16.579	0.00	0.00	0.13±0.33
12	2-Hexen-1-ol, acetate	10094-40-3	17.3	0.00	0.00	0.21±0.51
Ketone						
1	5-Hepten-2-one, 6-methyl-	110-93-0	15.432	0.68±0.34	0.88±0.4	1.05±0.36
2	1-Octen-3-one	4312-99-6	14.936	0.3±0.28	0.28±0.11	0.2±0.23

3	5, 9-Undecadien-2-one, 6, 10-dimethyl-, (E)-	3796-70-1	36.376	0.13±0.08	0.23±0.15	0.24±0.06
4	1-Decen-3-one	56606-79-2	14.983	0.00	0.00	0.18±0.29
Others						
1	Oxime-, methoxy-phenyl-_	67160-14-9	10.647	0.47±0.66	0.1±0.26	0.15±0.29
2	Furan, 2-pentyl-	3777-69-3	15.667	0.2±0.16	0.22±0.07	0.2±0.13
3	9-Oxabicyclo [6.1.0] nonane	286-62-4	6.991	0.28±0.68	0.00	0.00
4	4-Oxohex-2-enal	20697-55-6	13.932	0.00	0.13±0.2	0.00

Table S5. Identification of compounds in 'Jingbaili' pear by UPLC-MS/MS.

No.	Ion type	Formula	MS (m/z)	Fragment ions (m/z)	Identification	Structure types	RT (min)
1	$[M-H]^{-1}$	$C_6H_{14}O_6$	181.0714	181.0712, 71.0137, 101.0241	L-Iditol	Sugars	0.579
2	$[M-H]^{-1}$	$C_7H_{12}O_6$	191.0561	191.0564, 59.0139	D- (-)-Quinic acid	Organic acids	0.595
3	$[M+FA-H]^{-1}$	$C_{12}H_{22}O_{11}$	387.1132	341.1078, 89.0242, 179.0556	Sucrose	Sugars	0.597
4	$[M-H]^{-1}$	$C_4H_6O_5$	133.0141	115.0034, 133.0139, 71.0137	D- (+)-Malic acid	Organic acids	0.603
5	$[M-H]^{-1}$	$C_{16}H_{18}O_9$	353.0871	191.0557, 353.0867, 354.0886	Chlorogenic acid	Polyphenols	2.728

6	$[M-H]^{-1}$	$C_6H_{12}O_6$	179.0558	59.0137, 89.0243, 71.0138	D- (+)-Mannose	Sugars	0.588
7	$[M-H]^{-1}$	$C_9H_{10}O_3$	165.0556	147.0451, 119.0502, 72.9932	L-(-)-3-Phenyllactic acid	Organic acids	3.420
8	$[M-H]^{-1}$	$C_4H_4O_4$	115.0036	71.0137, 115.0034, 72.0171	Fumaric acid	Organic acids	0.604
9	$[M-H]^{-1}$	$C_4H_6O_3$	101.0242	101.0243, 100.9333	2-Oxobutyric acid	Organic acids	0.592
10	$[M-H]^{-1}$	$C_6H_{12}O_3$	131.0713	131.0713, 85.0659, 77.0021	6-Hydroxycaproic acid	Organic acids	3.209
11	$[M-H]^{-1}$	$C_9H_{16}O_4$	187.0975	187.0970, 125.0969, 169.0862	Azelaic acid	Organic acids	3.654

12	$[M-H]^{-1}$	$C_4H_8N_2O_3$	131.0462	131.0353, 88.0402, 41.9985	3-Ureidopropionic acid	Organic acids	0.570
13	$[M-H]^{-1}$	$C_6H_8O_7$	191.0194	111.0085, 87.0086, 191.0193	Citric acid	Organic acids	0.827
14	$[M-H]^{-1}$	$C_{18}H_{34}O_5$	329.2336	171.1030, 139.1131, 211.1339	(15Z)-9, 12, 13-Trihydroxy-15-octadecenoic acid	Organic acids	4.659
15	$[M-H]^{-1}$	$C_{15}H_{14}O_6$	289.0717	289.0707, 245.0813, 179.0341	Catechin	Polyphenols	2.963
16	$[M-H]^{-1}$	$C_6H_{12}O_7$	195.0509	195.0509, 75.0087, 129.0192	Gluconic acid	Organic acids	0.588
17	$[M-H]^{-1}$	$C_5H_7NO_3$	128.0353	128.0349	4-Oxoproline	Amino acids	0.612
18	$[M-H]^{-1}$	$C_{14}H_{22}O_2$	221.1545	221.1544, 222.1581	2, 5-di-tert-Butylhydroquinone	Polyphenols	6.335

19	$[M-H]^{-1}$	$C_7H_{10}O_5$	173.0456	93.0346, 111.0452, 173.0454	3, 4, 5-trihydroxycyclohex-1-ene-1-carboxylic acid	Organic acids	0.609
20	$[M-H]^{-1}$	$C_{27}H_{30}O_{16}$	609.1454	609.1448, 300.0269, 610.1483	Rutin	Polyphenols	3.167
21	$[M-H]^{-1}$	$C_9H_8O_3$	163.0400	162.8394, 119.0504, 163.0403	2-Hydroxycinnamic acid	Organic acids	3.205
22	$[M-H]^{-1}$	$C_{18}H_{32}O_5$	327.2182	327.2184, 328.2217	Corchorifatty acid F	Organic acids	4.356
23	$[M-H]^{-1}$	$C_{15}H_{20}O_4$	263.1288	153.0924, 219.1393, 263.1295	(±)-Abscisic acid	Organic acids	3.985
24	$[M-H]^{-1}$	$C_5H_{10}O_5$	149.0453	59.0136, 149.0003, 89.0242	D-(-)-Ribose	Sugars	0.593

25	$[M-H]^{-1}$	$C_6H_{10}O_6$	177.0403	99.0086, 129.0190, 59.0138	δ-Gluconic acid δ-lactone	Organic acids	0.600
26	$[M-H]^{-1}$	$C_5H_9NO_4$	146.0457	102.0560, 146.0458, 128.0353	N-Methyl-D-aspartic acid (NMDA)	Amino acids	0.600
27	$[M-H]^{-1}$	$C_{28}H_{32}O_{16}$	623.1630	623.1625, 315.0513, 314.0438	isorhamnetin 3-O-neohesperidoside	Polyphenols	3.326
28	$[M-H]^{-1}$	$C_8H_{14}O_4$	173.0819	111.0817, 173.0823, 57.0350	Suberic acid	Organic acids	3.310
29	$[M-H]^{-1}$	$C_{21}H_{20}O_{12}$	463.0878	463.0874, 300.0267, 301.0342	Quercetin-3β-D-glucoside	Polyphenols	3.261
30	$[M-H]^{-1}$	$C_6H_5NO_3$	138.0195	138.0196, 139.0231, 108.0216	6-Hydroxypicolinic acid	Organic acids	3.898
31	$[M-H]^{-1}$	$C_{21}H_{20}O_{11}$	447.0937	285.0408, 447.0927	Cynaroside	Polyphenols	3.479

32	$[M-H]^{-1}$	$C_{10}H_{10}O_4$	193.0506	134.0370, 193.0501	Ferulic acid	Polyphenols	3.400
33	$[M-H]^{-1}$	$C_{20}H_{30}N_2O_5$	377.2093	377.2089, 359.1992	Neotame	Others	6.461
34	$[M-H]^{-1}$	$C_4H_6O_4$	117.0192	73.0294, 117.0192, 116.9284	L-Threonic acid-1, 4-lactone	Organic acids	0.903
35	$[M-H]^{-1}$	$C_{15}H_{10}O_6$	285.0403	285.0411, 286.0444	Luteolin	Polyphenols	3.944
36	$[M-H]^{-1}$	$C_7H_6O_2$	121.0294	121.0291, 120.9909	4-Hydroxybenzaldehyde	Others	2.943
37	$[M-H]^{-1}$	$C_5H_4O_3$	111.0086	111.0085, 67.0188, 111.0186	2-Furoic acid	Organic acids	0.829
38	$[M-H]^{-1}$	$C_7H_6O_5$	169.0146	169.0145, 125.0247	Gallic acid	Polyphenols	1.033
39	$[M-H]^{-1}$	$C_{15}H_{20}O_8$	327.1085	165.0553, 230.8213, 115.6355	4-Acetyl-3-hydroxy-5-methylphenyl β-D-glucopyranoside	Sugars	2.624

40	$[M-H]^{-1}$	$C_9H_{14}O_2$	153.0921	153.0923, 138.0686, 104.9956	2-Norbornaneacetic acid	Organic acids	3.985
41	$[M-H]^{-1}$	$C_{30}H_{44}O_9$	547.2913	547.2924, 503.3020	Cymarin	Others	3.621
42	$[M-H]^{-1}$	$C_4H_6O_4$	117.0191	116.9283, 73.0294, 59.0137	Methylmalonic acid	Organic acids	0.625
43	$[M-H]^{-1}$	$C_7H_6O_3$	137.0245	137.0241, 92.9200	Salicylic acid	Polyphenols	2.682
44	$[M-H]^{-1}$	$C_{17}H_{20}O_9$	367.1018	191.0557, 193.0499	3-O-Feruloylquinic acid	Polyphenols	3.107
45	$[M-H]^{-1}$	$C_{11}H_{20}O_4$	215.1288	215.1291, 197.1186, 153.1288	Undecanedioic acid	Organic acids	4.388
46	$[M-H]^{-1}$	$C_{21}H_{20}O_{11}$	447.0929	447.0631, 284.0325	Trifolin	Polyphenols	3.409
47	$[M-H]^{-1}$	$C_{21}H_{23}FN_2O_3S$	401.1357	401.1358, 369.1098	Besonprodil	Others	3.998
48	$[M-H]^{-1}$	$C_5H_8O_5$	147.0298	61.9885, 147.0303	δ-Ribono-1, 4-lactone	Sugars	0.900

49	$[M-H]^{-1}$	$C_8H_{15}NO_3$	172.0980	172.0983, 111.0817	Hexanoylglycine	Amino acids	2.782
50	$[M-H]^{-1}$	$C_{16}H_{22}O_{10}$	373.1136	211.0608, 373.1139	Geniposidic acid	Organic acids	2.407
51	$[M-H]^{-1}$	$C_6H_{10}O_4$	145.0506	145.0509, 101.0610, 83.0502	Adipic acid	Organic acids	2.339
52	$[M-H]^{-1}$	$C_{17}H_{24}O_{10}$	387.1316	207.0668, 101.0521	Geniposide	Others	2.806
53	$[M-H]^{-1}$	$C_{12}H_{22}O_4$	229.1447	229.1449, 211.1344	Dodecanedioic acid	Organic acids	4.944
54	$[M-H]^{-1}$	$C_{13}H_{10}O_3$	213.0561	93.0348, 213.0560	4, 4'-Dihydroxybenzophenone	Others	5.230
55	$[M-H]^{-1}$	$C_{21}H_{20}O_{10}$	431.0986	431.0983, 269.0464	Apigetrin	Polyphenols	3.485
56	$[M-H]^{-1}$	$C_{18}H_{32}O_4$	311.2224	311.2213, 211.1338	(±)9-HpODE	Others	6.795

57	$[M-H]^{-1}$	$C_7H_{13}NO_3$	158.0821	158.0823, 114.0927, 157.8631	N-Isovalerylglycine	Amino acids	3.161
58	$[M-H]^{-1}$	$C_{12}H_{18}O_3$	209.1183	59.0139, 188.9973, 209.1193	Jasmonic acid	Organic acids	4.525
59	$[M-H]^{-1}$	$C_{11}H_{12}N_2O_2$	203.0829	203.0823, 116.0506, 74.0248	D- (+)-Tryptophan	Amino acids	2.556
60	$[M-H]^{-1}$	$C_6H_6O_6$	173.0090	85.0293, 111.0085, 173.0447	trans-Aconitic acid	Organic acids	0.828
61	$[M-H]^{-1}$	$C_{25}H_{24}O_{12}$	515.1194	353.0884, 191.0566	4, 5-Dicaffeoylquinic acid	Organic acids	3.421
62	$[M+FA-H]^{-1}$	$C_{15}H_{20}O_9$	389.1081	181.0512, 343.1044	3-[3-(beta-D-Glucopyranosyloxy)-2-hydroxyphenyl] propanoic acid	Organic acids	2.588

63	$[M-H]^{-1}$	$C_9H_{12}N_2O_6$	243.0628	243.0635, 110.0249, 200.0572	Uridine	nucleotides	0.830
64	$[M+H]^{+1}$	$C_{13}H_{13}N_3$	212.1177	212.1178, 119.0601	N, N'-Diphenylguanidine	Others	3.267
65	$[M+H]^{+1}$	$C_{13}H_{24}N_2O$	225.1957	225.1959, 100.1120	N, N'-Dicyclohexylurea	Others	5.552
66	$[M+H]^{+1}$	$C_{20}H_{21}N_3O_3$	352.1650	352.1648, 212.1179	2-[1-(4-isobutylphenyl) ethyl]-5-(3-nitrophenyl)-1, 3, 4-oxadiazole	Others	3.653
67	$[M+H]^{+1}$	$C_{13}H_{12}N_2O$	213.1019	213.1020, 94.0650	N, N'-Diphenylurea	Others	5.346
68	$[M+H]^{+1}$	$C_9H_{18}N_2O$	171.1489	171.1491, 89.0708	N-cyclooctylurea	Others	3.880
69	$[M+H]^{+1}$	$C_7H_8N_2O$	137.0707	137.0708, 94.0650	3-Aminobenzamide	Amino acids	2.970

70	$[M+H]^{+1}$	$C_{13}H_{18}N_2O$	219.1488	219.1490, 94.0650, 137.0707	Acetyl norfentanyl	Others	5.443
71	$[M+H]^{+1}$	$C_{19}H_{23}N_3$	294.1960	294.1960, 212.1180	Amitraz	Others	4.915
72	$[M+H]^{+1}$	$C_{18}H_{39}NO_3$	318.2995	318.2995, 256.2629	2-Amino-1, 3, 4-octadecanetriol	Others	6.440
73	$[M+H]^{+1}$	$C_9H_{19}NO$	158.1536	158.1535, 102.0910	2, 2, 6, 6-Tetramethyl-4-piperidinol	Others	5.181
74	$[M+H]^{+1}$	$C_{14}H_{15}N$	198.1273	198.1273, 91.0540	Dibenzylamine	Others	3.451
75	$[M+H]^{+1}$	$C_7H_5NS_2$	167.9934	167.9934	2-Mercaptobenzothiazole	Others	4.359
76	$[M+H]^{+1}$	$C_{15}H_{17}N_3$	240.1492	240.1493, 195.0915	1, 3-di-o-Tolylguanidine	Others	3.722
77	$[M+H]^{+1}$	$C_{20}H_{23}NO_4$	342.1707	342.1709, 148.0868	6-Acetylcodeine	Others	4.837

78	$[M+H]^{+1}$	$C_{15}H_{15}NO$	226.1222	226.1226, 91.0542, 148.0757	4-(Dimethylamino)benzophenone	Others	6.133
79	$[M+H]^{+1}$	$C_{13}H_{21}NO_3$	240.1591	240.1591, 158.0810	Salbutamol	Others	4.395
80	$[M+H]^{+1}$	C_7H_5NOS	152.0163	152.0164, 153.0196	2-Hydroxybenzothiazole	Others	3.932
81	$[M+H]^{+1}$	$C_8H_{19}N$	130.1588	130.1587, 57.0698, 74.0962	N, N-Diisopropylethylamine (DIPEA)	Others	2.600
82	$[M+H]^{+1}$	$C_7H_{13}NO$	128.1069	128.1068, 46.0287, 83.0854	N-Methylcaprolactam	Others	3.484
83	$[M+H]^{+1}$	$C_5H_{13}NO$	104.1068	104.1067, 105.1102	Choline	Others	0.575
84	$[M+H]^{+1}$	$C_7H_8O_3$	141.0545	113.0596, 81.0334, 141.0545	2-Methoxyresorcinol	Others	4.258

85	$[M+H+MeOH]^{+1}$	$C_{12}H_{18}N_2O$	239.1750	207.1495, 164.1066, 124.0760	N'-Hydroxy-4-pentylbenzenecarboximidamide	Others	2.931
86	$[M+H]^{+1}$	$C_{13}H_{22}N_2O_2$	239.1749	239.1747, 207.1487, 189.1382	1, 5-Ditetrahydro-1H-pyrrol-1-ylpentane-1, 5-dione	Others	2.928
87	$[M+H]^{+1}$	$C_4H_8N_2O_3$	133.0606	87.0551, 133.0605, 74.0235	Asparagine	Amino acids	0.583
88	$[M+H]^{+1}$	$C_{11}H_9NO_2$	188.0704	145.0599, 188.0704, 144.0806	Indole-3-acrylic acid	Organic acids	2.550
89	$[M+H-NH3]^{+1}$	$C_{11}H_{12}N_2O_2$	188.0703	188.0704, 146.0599	L-Tryptophan	Amino acids	2.550
90	$[M+H]^{+1}$	$C_4H_{11}NO_2$	106.0860	106.0859, 88.0754, 70.0650	Diethanolamine	Others	0.577
91	$[M+H+MeOH]^{+1}$	$C_6H_6O_3$	159.0650	99.0439, 81.0337	4-Hydroxy-6-methyl-2-pyrone	Others	4.260

92	$[M+H]^{+1}$	$C_{12}H_{14}N_4O_4S_2$	343.0525	151.0323, 343.0527, 311.0265	Thiophanate-methyl	Others	4.454
93	$[M+H]^{+1}$	$C_{18}H_{35}NOS_2$	346.2220	346.2229, 101.0595, 147.2208	dodecyl [(tetrahydrofuran-2-ylmethyl) amino] methanedithioate	Others	3.871
94	$[M+H]^{+1}$	$C_9H_9N_3O_2$	192.0765	192.0765, 160.0503	Carbendazim	Others	2.671
95	$[M+H]^{+1}$	$C_{15}H_{18}N_2O_2$	259.1439	259.1433, 177.0657	4, 4'-Propane-2, 2-diylbis(2-aminophenol)	Others	5.698
96	$[M+H]^{+1}$	$C_{10}H_{13}N_5O_4$	268.1037	136.0615, 268.1036	Adenosine	nucleotides	0.834
97	$[M+H]^{+1}$	$C_{20}H_{21}NO_4$	340.1553	340.1564, 341.1591, 146.0712	Papaverine	Others	4.041

98	$[M+H]^{+1}$	$C_9H_6O_3$	163.0388	163.0388, 164.0422	4-Hydroxycoumarin	Others	5.256
99	$[M+H]^{+1}$	$C_{12}H_{27}OP$	219.1870	219.1871, 220.1905	Tributylphosphine oxide	Others	5.738
100	$[M+H]^{+1}$	$C_{13}H_{25}N$	196.2058	196.2058, 197.2092, 114.1277	N-Cyclohexyl-N-methylcyclohexanamine	Others	3.592
101	$[M+H]^{+1}$	$C_{15}H_{13}N_3O$	252.1128	252.1126, 106.0649, 59.0491	7-Aminonitrazepam	Others	3.503
102	$[M+H]^{+1}$	$C_{10}H_{10}O_4$	195.0650	163.0390, 195.1374	Dimethyl phthalate	Others	4.532
103	$[M+H]^{+1}$	$C_9H_{17}NO_2$	172.1331	172.1330, 90.0548	Gabapentin	Others	3.594
104	$[M+H]^{+1}$	$C_{19}H_{21}N_3O$	308.1754	308.1756, 86.0964	Zolpidem	Others	4.349

105	$[M+H]^{+1}$	$C_9H_{13}N_3$	164.1180	164.1180, 46.0651	N1-[2-(4-Pyridyl) ethyl] ethanimidamide	Others	2.273
106	$[M+H]^{+1}$	$C_{21}H_{27}NO$	310.2161	310.2169, 211.1116, 133.0646	Methadone	Others	5.100
107	$[M+H]^{+1}$	$C_{12}H_{27}NO$	202.2163	202.2163, 203.2192	N, N-Dimethyldecylamine N-oxide	Others	4.768
108	$[M+H]^{+1}$	$C_{22}H_{47}NO_5$	406.3522	406.3516, 300.2887, 256.2637	Hydrolyzed fumonisin B1	Others	6.549
109	$[M+H]^{+1}$	$C_{18}H_{19}N_3O$	294.1597	294.1599, 72.0807, 295.1632	Ondansetron	Others	3.959
110	$[M+H]^{+1}$	$C_{19}H_{18}N_4O_2$	335.1500	335.1499, 336.1530	{(3S)-3-[5-(4-Methylphenyl)-1,3,4-oxadiazol-2-yl]-1-pyrrolidinyl}(2-pyridinyl) methanone	Others	6.002

111	$[M+H]^{+1}$	$C_{13}H_{21}NO_2$	224.1643	224.1643, 142.0861, 99.0803	Toliprolol	Others	5.427
112	$[M+H]^{+1}$	$C_8H_6O_2$	135.0438	135.0443, 136.0218	Phthaldialdehyde	Others	4.547
113	$[M+H]^{+1}$	$C_{10}H_{15}NO_2$	182.1173	182.1173, 164.1068, 82.0650	N-Phenyldiethanolamine	Others	1.041
114	$[M+H]^{+1}$	$C_{13}H_{25}NO_4$	260.1853	260.1852, 88.0755, 199.1325	Hexanoylcarnitine	Others	3.236
115	$[M+H]^{+1}$	$C_6H_{15}NO_3$	150.1121	150.1121, 88.0754	Triethanolamine	Others	0.589
116	$[M+H]^{+1}$	$C_8H_{15}NO$	142.1225	142.1225, 60.0444, 83.0854	3, 3, 5, 5-Tetramethylpyrroline-N-oxide	Others	3.535
117	$[M+H]^{+1}$	$C_{15}H_{15}N_3O_2$	270.1234	270.1233, 271.1266	Methyl red	Others	2.688

118	$[M+H]^{+1}$	$C_{12}H_{23}NO$	198.1850	198.1852, 142.1226	Laurolactam	Others	5.447
119	$[M+H]^{+1}$	$C_{10}H_{12}O_2$	165.0908	165.0908, 147.0803, 119.0854	4-Phenylbutyric acid	Organic acids	3.219
120	$[M+H]^{+1}$	$C_{11}H_{20}N_2O$	197.1647	197.1647, 72.0807, 98.0599	4-heptyl-3-methyl-1H-pyrazol-5-ol	Others	4.311
121	$[M+H]^{+1}$	$C_6H_6N_2O$	123.0552	123.0551, 124.0755	Nicotinamide	vitamins	0.834
122	$[M+H]^{+1}$	$C_5H_9NO_2$	116.0704	116.0705, 70.0651	Proline	Amino acids	0.601
123	$[M+H]^{+1}$	$C_9H_{21}NO_3$	192.1591	192.1589, 174.1485, 156.1381	Triisopropanolamine	Others	0.608
124	$[M+H]^{+1}$	$C_7H_7NO_2$	138.0548	138.0546, 139.0482	Anthranilic acid	Organic acids	0.600

125	$[M+H]^{+1}$	$C_{10}H_{21}NO$	172.1694	172.1691, 171.1488, 116.1068	Decanamide	Others	5.317
126	$[M+H]^{+1}$	$C_9H_{11}NO_2$	166.0861	120.0806, 166.0860	L-Phenylalanine	Amino acids	1.505
127	$[M+H]^{+1}$	$C_5H_{11}NO_2$	118.0861	118.0859, 72.0806	Valine	Amino acids	0.600
128	$[M+H]^{+1}$	$C_{16}H_{25}NO_4$	296.1852	296.1851, 264.1594	Esmolol	Others	3.249
129	$[M+H]^{+1}$	$C_8H_8O_3$	153.0545	153.0545, 125.0596	Vanillin	Others	3.332
130	$[M+H]^{+1}$	$C_8H_8O_4$	169.0493	169.0494, 141.0545, 109.0284	Vanillic acid	Organic acids	2.978
131	$[M+H]^{+1}$	$C_{10}H_{15}NO_2$	182.1173	182.1171, 159.0127, 82.0649	Anhydroecgonine methyl ester	Others	0.835

132	$[M+H]^{+1}$	$C_{10}H_8O_3$	177.0544	177.1649, 149.0232	4-Methylumbelliferone hydrate	Others	6.13
133	$[M+H]^{+1}$	$C_{21}H_{22}O_8$	403.1384	403.1389, 373.0916	Nobiletin	Polyphenols	5.715
134	$[M+H]^{+1}$	$C_{10}H_{17}NO_3$	200.1279	200.1278, 182.1173	Ecgonine methyl ester	Others	0.836
135	$[M+H]^{+1}$	$C_6H_{13}NO_2$	132.1018	86.0963, 132.1018	Isoleucine	Amino acids	0.939
136	$[M+H]^{+1}$	$C_{10}H_{13}NO_2$	180.1017	180.1018, 162.0911	Phenacetin	Organic acids	1.113
137	$[M+H]^{+1}$	$C_6H_9N_3O_2$	156.0765	110.0711, 156.0766	L-Histidine	Amino acids	0.580
138	$[M+H]^{+1}$	$C_{18}H_{19}N_3O$	294.1598	294.1603, 293.2215, 72.0807	Fabesetron	Others	4.006

139	$[M+H]^{+1}$	$C_8H_{17}NO_2$	160.1330	160.1330, 115.0752, 55.0542	Pregabalin	Organic acids	0.614
140	$[M+H]^{+1}$	$C_6H_6N_2O_2$	139.0500	139.0500, 121.0393	Urocanic acid	Organic acids	0.607
141	$[M+H]^{+1}$	$C_{11}H_9NO_2$	188.0704	188.0705, 146.0599, 187.1266	trans-3-Indoleacrylic acid	Organic acids	1.007
142	$[M+H]^{+1}$	$C_9H_{10}N_2$	147.0916	147.0918, 120.0443	5, 6-Dimethylbenzimidazole	vitamins	3.722
143	$[M+H]^{+1}$	$C_{28}H_{31}FN_4O$	459.2559	459.2556, 460.2593	Astemizole	Others	4.401
144	$[M+H]^{+1}$	$C_{16}H_{13}N_3O$	264.1129	264.1130, 265.1150, 146.0600	4-(4-Methoxyphenyl)-2-(4-pyridinyl) pyrimidine	Others	3.672
145	$[M+H]^{+1}$	$C_5H_9NO_4$	148.0601	69.0334, 130.0495, 148.0598	L-Glutamic acid	Amino acids	0.588

146	$[M+H]^{+1}$	$C_{12}H_{19}N$	178.1589	178.1590, 179.0637	2-Ethylamino-1-phenylbutane	Others	6.718
147	$[M+H]^{+1}$	$C_{11}H_{15}N_5O_3S$	298.0964	136.0614, 298.0958	5'-S-Methyl-5'-thioadenosine	nucleotides	2.471
148	$[M+H]^{+1}$	$C_6H_{13}NO_2$	132.1018	86.0962, 132.1017	L-Norleucine	Amino acids	1.000
149	$[M+H]^{+1}$	$C_{14}H_{20}N_6O_5S$	385.1279	385.1281, 307.0965	S-Adenosylhomocysteine	Amino acids	4.139
150	$[M+H]^{+1}$	$C_{18}H_{15}OP$	279.0929	279.0930, 280.0965, 149.0229	Triphenylphosphine oxide	Others	5.413
151	$[M+H]^{+1}$	$C_{18}H_{30}O_2$	279.2317	149.0233, 279.0933, 81.0700	α-Eleostearic acid	Organic acids	0.820
152	$[M+H]^{+1}$	$C_4H_7NO_4$	134.0444	134.0445, 74.0235, 87.0550	L-Aspartic acid	Amino acids	0.589

153	$[M+H]^{+1}$	$C_{28}H_{34}O_{15}$	611.1966	303.0861, 611.1949	Hesperidin	Polyphenols	3.483
154	$[M+H]^{+1}$	$C_{7}H_{17}N$	116.1433	116.1430, 46.0650	Butylisopropylamine	Others	1.357
155	$[M+H]^{+1}$	$C_{18}H_{19}NO$	266.1535	266.1537, 91.0540, 224.1432	Desmethyldoxepin	Others	6.264
156	$[M+H]^{+1}$	$C_{5}H_{7}NO_{3}$	130.0497	130.0498, 84.0443, 86.0964	L-Pyroglutamic acid	Amino acids	0.834
157	$[M+H]^{+1}$	$C_{12}H_{17}NO_{2}$	208.1329	208.1330, 89.0595, 209.1367	MDMA Methylene homolog	Others	2.545
158	$[M+H]^{+1}$	$C_{20}H_{20}O_{7}$	373.1279	373.1282, 343.0810	Tangeritin	Polyphenols	6.458
159	$[M+H]^{+1}$	$C_{6}H_{14}N_{4}O_{2}$	175.1187	175.1188, 116.0705, 60.0556	DL-Arginine	Amino acids	0.574
160	$[M+H]^{+1}$	$C_{11}H_{12}O_{4}$	209.0807	209.0805, 109.0646, 149.0596	(2E)-3-(3, 4-dimethoxyphenyl) prop-2-enoic acid	Organic acids	4.152

161	$[M+H]^{+1}$	$C_{12}H_{22}O_{11}$	343.1223	344.0885, 127.0386, 145.0493	D-Lactose monohydrate	Sugars	0.587
162	$[M+H]^{+1}$	$C_{15}H_{14}O_{6}$	291.0859	139.0386, 123.0438, 291.0842	Epicatechin	Polyphenols	2.962
163	$[M+H]^{+1}$	$C_{12}H_{17}NO$	192.1380	192.1379, 133.0645	N-Ethyl-N-methylcathinone	Others	1.748
164	$[M+H]^{+1}$	$C_{6}H_{12}N_{4}$	141.1133	141.1134, 112.0869	Hexamethylenetetramine	Others	0.584
165	$[M+H]^{+1}$	$C_{12}H_{27}N$	186.2213	186.2212, 187.1267, 57.0698	Dihexylamine	Others	6.525
166	$[M+H]^{+1}$	$C_{9}H_{7}NO$	146.0599	146.0600, 86.0964	4-Indolecarbaldehyde	Others	2.551
167	$[M+H]^{+1}$	$C_{19}H_{17}N_{3}O$	304.1441	304.1442, 305.1451	(+)-Evodiamine	Others	3.944
168	$[M+H]^{+1}$	$C_{5}H_{5}N_{5}$	136.0616	136.0617, 137.1326	Adenine	Others	0.834

169	$[M+H]^{+1}$	$C_{14}H_{20}N_6O_5S$	385.1279	385.1278, 307.0964	S-Adenosyl-L-homocysteine	Amino acids	4.247
170	$[M+H+MeOH]^{+1}$	$C_{10}H_{14}N_2$	195.1491	163.1228, 132.0806	Nicotine	Others	0.832
171	$[M+H]^{+1}$	$C_{12}H_{25}NO_2$	216.1955	216.1955, 198.1849	12-Aminododecanoic acid	Organic acids	3.912
172	$[M+H]^{+1}$	$C_{12}H_{16}O_3$	209.1169	209.1168, 135.0802	β-Asarone	Others	4.076
173	$[M+H]^{+1}$	$C_8H_{12}N_2O$	153.1022	153.1020, 129.0307, 58.0652	6-(tert-butyl) pyridazin-3(2H)-one	Others	0.614
174	$[M+H]^{+1}$	$C_{12}H_{19}NO_3$	226.1436	226.1427, 227.1757	Terbutaline	Others	2.459
175	$[M+H]^{+1}$	$C_{21}H_{21}N$	288.1742	288.1740, 91.0540, 181.1004	Tribenzylamine	Others	5.161
176	$[M+H]^{+1}$	$C_{10}H_{10}O$	147.0803	147.0803, 119.0853	4-Phenyl-3-buten-2-one	Others	2.856

177	$[M+H]^{+1}$	$C_{10}H_{11}NO$	162.0912	139.9820, 162.0912, 163.0390	3-(2-Hydroxyethyl) indole	Others	1.235
178	$[M+H]^{+1}$	$C_9H_{10}O_3$	167.0701	167.0701, 84.9596	Apocynin	Others	3.779
179	$[M+H]^{+1}$	$C_{10}H_{16}O$	153.1272	153.1270, 71.0492	Citral	Others	3.721
180	$[M+H]^{+1}$	$C_8H_8O_2$	137.0596	137.0597, 137.0959	4-Methoxybenzaldehyde	Others	3.789
181	$[M+H]^{+1}$	$C_{15}H_{23}NO_4$	282.1698	282.1705, 89.0597	Cycloheximide	Others	2.256
182	$[M+H]^{+1}$	C_7H_9N	108.0806	108.0804, 109.0313	o-Toluidine	Others	0.605
183	$[M+H]^{+1}$	C_7H_5NS	136.0214	136.0215, 135.0804	Benzothiazole	Others	5.206
184	$[M+H]^{+1}$	$C_{11}H_{20}N_2O_3$	229.1539	229.1542, 230.1585	Leucylproline	Amino acids	0.610
185	$[M+H]^{+1}$	$C_{27}H_{30}O_{16}$	611.1592	303.0492, 611.2062	Quercetin 3-O-rhamnoside-7-O-glucoside	Polyphenols	3.162

186	$[M+H]^{+1}$	$C_8H_{17}NO$	144.1380	144.1380, 88.0755	Valpromide	Others	4.520
187	$[M+H]^{+1}$	C_7H_5NOS	152.0163	152.0163, 124.1119	Benzothiazolone	Others	3.235
188	$[M+H]^{+1}$	$C_{20}H_{42}O_6$	379.3049	73.0647, 145.1221	Poly THF n5	Others	4.493
189	$[M+H]^{+1}$	$C_{10}H_{10}O$	147.0804	147.0803, 123.9642	Benzylideneacetone	Others	2.740
190	$[M+H]^{+1}$	$C_7H_{15}NO_2$	146.1172	146.1171, 86.0962, 69.0333	Acetylcholine	Others	0.610
191	$[M+H]^{+1}$	$C_{11}H_{15}NO_2$	194.1173	194.1174, 162.0913, 120.0808	4-(Diethylamino)salicylaldehyde	Others	2.788
192	$[M+H]^{+1}$	$C_{17}H_{19}N_3O_2$	298.1546	298.1549, 195.0916, 104.0705	1-benzyl-4-(4-nitrophenyl) piperazine	Others	3.828
193	$[M+H]^{+1}$	$C_{12}H_{27}N$	186.2214	186.2212, 187.2245	Tributylamine	Others	4.250

194	$[M+H]^{+1}$	$C_{10}H_{22}O_4$	207.1588	207.1863, 89.0596	Triethylene glycol monobutyl ether	Others	3.800
195	$[M+H]^{+1}$	$C_{13}H_{18}O$	191.1429	191.1427, 109.1011, 135.0805	Heptanophenone	Others	4.163
196	$[M+H]^{+1}$	$C_9H_{11}NO_3$	182.0810	136.0756, 165.0545, 123.0440	L-Tyrosine	Amino acids	0.611
197	$[M+H]^{+1}$	$C_9H_{15}NO_3$	186.1122	186.1120, 187.1263, 88.0216	Ecgonine	Others	1.220
198	$[M+H]^{+1}$	$C_4H_9N_3O_2$	132.0766	132.0765, 86.0962, 74.0235	Creatine	Organic acids	0.595
199	$[M+H]^{+1}$	$C_4H_{11}O_4P$	155.0466	98.9840, 113.9636, 154.9902	Diethyl phosphate	Others	3.784

200	$[M+H]^{+1}$	$C_9H_{19}NO_4$	206.1385	206.1389, 76.0756, 188.1279	D-Panthenol	vitamins	1.793
201	$[M+H]^{+1}$	$C_{11}H_{15}NO$	178.1223	178.1224, 179.0642	Phenmetrazine	Others	6.873
202	$[M+H]^{+1}$	$C_{16}H_{24}N_2O_2$	277.1911	277.1904, 245.1643	Norsufentanil	Others	3.051
203	$[M+H]^{+1}$	$C_{15}H_{10}O_7$	303.0494	303.0493, 304.0532	Quercetin	Polyphenols	3.224
204	$[M+ACN+H]^{+1}$	C_8H_7N	159.0916	119.0853, 91.0540	Indole	Others	2.550
205	$[M+H]^{+1}$	$C_{13}H_{20}N_2O$	221.1647	221.1646, 189.1396	N-(tert-butyl)-N'-phenethylurea	Others	0.925
206	$[M+H]^{+1}$	$C_9H_{21}N$	144.1744	144.1744, 145.1778	N-Methyloctan-1-amine	Others	3.064
207	$[M+H]^{+1}$	$C_{11}H_{16}N_2O$	193.1333	193.1338, 112.0756	1-(4-Anisyl) piperazine	Others	0.830

208	$[M+H]^{+1}$	$C_6H_8N_2O$	125.0708	125.0707, 110.0473	4, 6-dimethylpyrimidin-2-ol	Others	0.612
209	$[M+H]^{+1}$	$C_7H_{13}NO_2$	144.1017	144.1016, 85.0282	DL-Stachydrine	Others	0.611
210	$[M+H]^{+1}$	$C_{12}H_{16}N_4OS$	265.1116	122.0711, 265.1117	Thiamine	vitamins	0.814
211	$[M+H]^{+1}$	$C_{17}H_{19}NO_3$	286.1434	91.0542, 286.1445	Morphine	Others	6.023
212	$[M+H]^{+1}$	$C_{15}H_{14}O_5$	275.0910	275.0905, 107.0490	Phloretin	Polyphenols	3.713
213	$[M+H]^{+1}$	$C_9H_{10}O$	135.0803	135.0802, 107.0852	2, 4-Dimethylbenzaldehyde	Others	3.580
214	$[M+H]^{+1}$	$C_9H_{21}N$	144.1745	144.1745, 121.9659	Octylmethylamine	Others	3.926
215	$[M+H]^{+1}$	$C_{10}H_8O_3$	177.0544	149.0233, 176.1519	hymecromone	Polyphenols	5.256
216	$[M+H]^{+1}$	$C_{15}H_{18}N_2$	227.1539	184.0994, 227.1539	N-Isopropyl-N'-phenyl-p-phenylenediamine	Others	3.890

217	$[M+H]^{+1}$	$C_9H_{20}N_2O_2$	189.1594	189.1592, 130.0861, 84.0807	N6, N6, N6-Trimethyl-L-lysine	Amino acids	0.552
218	$[M+H]^{+1}$	$C_{17}H_{27}NO_4$	310.2008	310.1997, 311.2019	Nadolol	Others	2.898
219	$[M+H]^{+1}$	$C_6H_8N_2$	109.0759	109.0757, 82.0649	2, 5-Dimethylpyrazine	Others	0.850
220	$[M+H]^{+1}$	$C_{15}H_{15}NO_3$	258.1122	258.1110, 230.1160	Haplamine	Others	4.637
221	$[M+H]^{+1}$	$C_8H_{12}N_2$	137.1073	137.1073, 137.0966	Tetramethylpyrazine	Others	3.676
222	$[M+H]^{+1}$	$C_9H_{10}ClN_5O_2$	256.0595	256.2632, 209.0586, 175.0978	Imidacloprid	Others	3.557
223	$[M+H]^{+1}$	$C_{13}H_{20}N_2O_2$	237.1596	237.1597, 219.1506	Levodropropizine	Others	4.791
224	$[M+H]^{+1}$	C_4H_8OS	105.0368	105.0367, 61.0106	Tetramethylene sulfoxide	Others	0.948

225	$[M+H]^{+1}$	$C_9 H_{13} N$	136.1119	136.0758, 91.0542	2-Isopropylaniline	Others	1.089
226	$[M+H]^{+1}$	$C_{16} H_{12} O_7$	317.0654	317.0648, 318.0692	Rhamnetin	Polyphenols	3.427
227	$[M+H]^{+1}$	$C_{10} H_{15} N O$	166.1224	166.1223, 84.9596	Perillartine	Others	5.208
228	$[M+H]^{+1}$	$C_{22} H_{28} N_2 O_2$	353.2224	353.2215, 354.2262	β-Hydroxyfentanyl	Others	4.800
229	$[M+H]^{+1}$	$C_{15} H_{18} Cl N_3 O$	292.1216	292.1217, 70.0399, 291.1186	Uniconazole	Others	6.926
